# Self-Assembly and Cytocompatibility of Amino Acid Conjugates Containing a Novel Water-Soluble Aromatic Protecting Group

**DOI:** 10.1021/acs.biomac.3c00860

**Published:** 2023-11-01

**Authors:** Valeria Castelletto, Lucas de Mello, Emerson Rodrigo da Silva, Jani Seitsonen, Ian W Hamley

**Affiliations:** † School of Chemistry, Food Biosciences and Pharmacy, University of Reading, Whiteknights, Reading RG6 6AD, United Kingdom; ‡ Departamento de Biofísica, Universidade Federal de São Paulo, São Paulo 04023-062, Brazil; § Nanomicroscopy Center, Aalto University, Puumiehenkuja 2, FIN-02150 Espoo, Finland

## Abstract

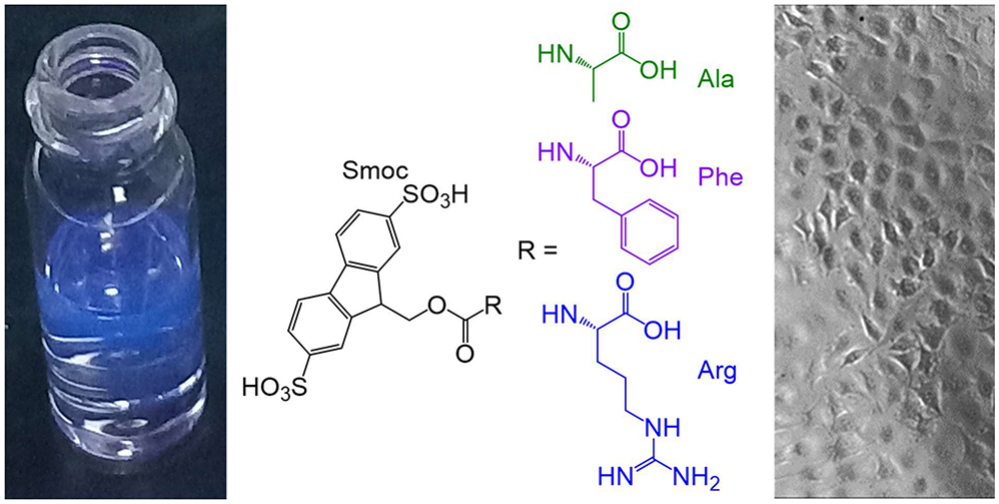

There has been considerable interest in peptides in which the Fmoc (9-fluorenylmethoxycarbonyl) protecting group is retained at the N-terminus, since this bulky aromatic group can drive self-assembly, and Fmoc-peptides are biocompatible and have applications in cell culture biomaterials. Recently, analogues of new amino acids with 2,7-disulfo-9-fluorenylmethoxycarbonyl (Smoc) protecting groups have been developed for water-based peptide synthesis. Here, we report on the self-assembly and biocompatibility of Smoc-Ala, Smoc-Phe and Smoc-Arg as examples of Smoc conjugates to aliphatic, aromatic, and charged amino acids, respectively. Self-assembly occurs at concentrations above the critical aggregation concentration (CAC). Cryo-TEM imaging and SAXS reveal the presence of nanosheet, nanoribbon or nanotube structures, and spectroscopic methods (ThT fluorescence circular dichroism and FTIR) show the presence of β-sheet secondary structure, although Smoc-Ala solutions contain significant unaggregated monomer content. Smoc shows self-fluorescence, which was used to determine CAC values of the Smoc-amino acids from fluorescence assays. Smoc fluorescence was also exploited in confocal microscopy imaging with fibroblast cells, which revealed its uptake into the cytoplasm. The biocompatibility of these Smoc-amino acids was found to be excellent with zero cytotoxicity (in fact increased metabolism) to fibroblasts at low concentration.

## Introduction

**Scheme 1:**
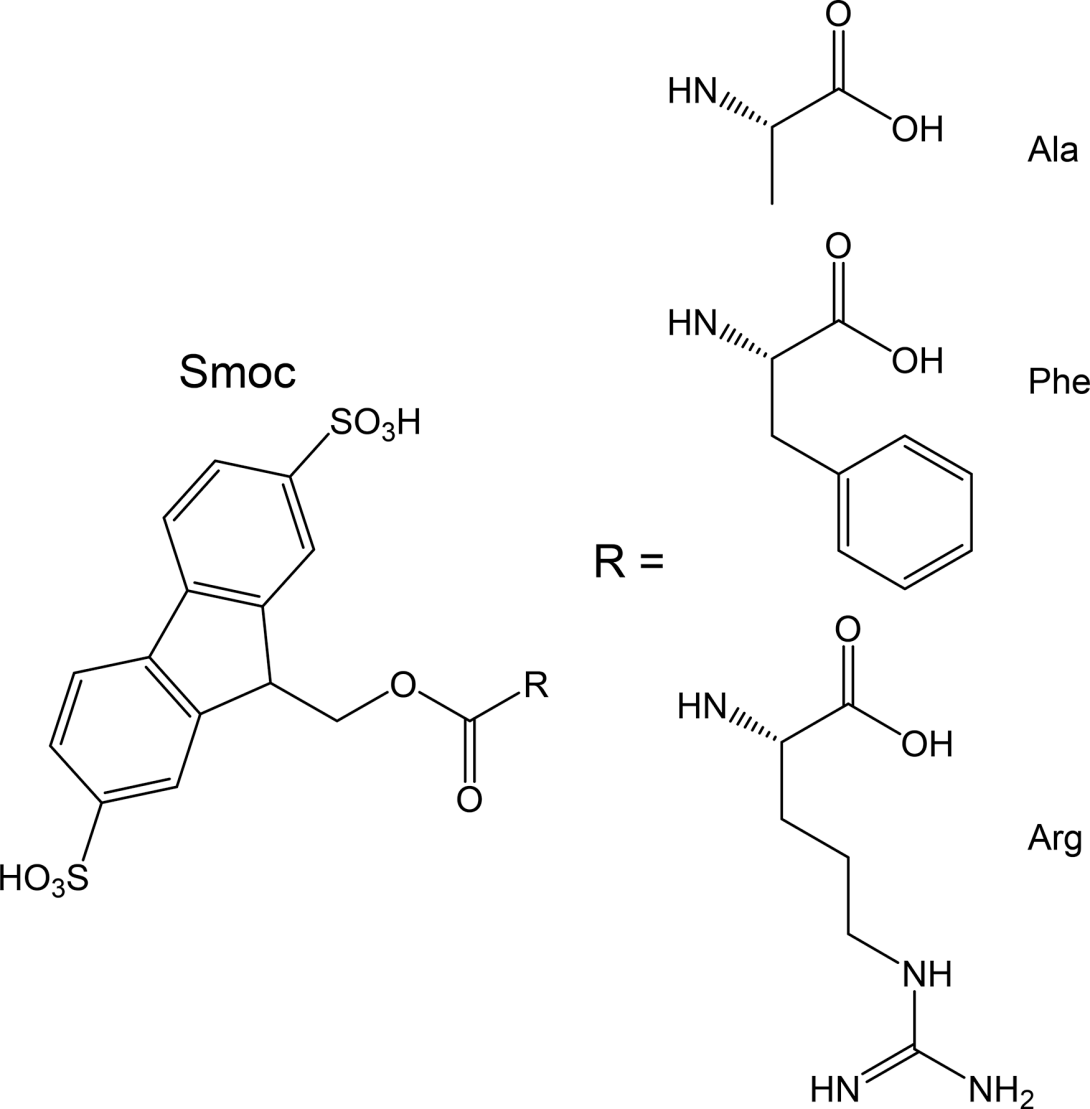
Schematic of Smoc-Amino Acids Studied: Smoc-Ala, Smoc-Phe, and Smoc-Arg

Conjugation of peptides or even amino acids to bulky terminal groups is a powerful tool to impart self-assembly behavior, for example, fibril formation.^1−6^ This topic has recently been reviewed.^7^ Self-assembly in such systems is often driven by π–π stacking interactions of bulky aromatic groups, such as Fmoc (9-fluorenylmethoxycarbonyl). This in turn leads to a diversity of properties, such as hydrogelation, and this can further be used in the development of cell culture materials. Good cytocompatibility is possible with the correct choice of the constituent peptides/amino acids. The main application of Fmoc-amino acids is, of course, in solid phase synthesis via sequential protection/deprotection reactions. The Fmoc group is hydrophobic, so synthesis is carried out in organic solvents. There is great interest in reducing the environmental impact of organic solvents used in synthetic reactions, in peptide synthesis, and other fields. Many approaches to cleaner peptide synthesis have been investigated.^8−11^ Recently, the Fmoc group has been modified with sulfonic acid groups to confer water solubility, hence enabling sustainable green synthesis of peptides in aqueous media.^12,13^ This follows on from Merrifield’s original development of monosulfated 2-sulfo-9-fluorenylmethyloxycarbonyl chloride amino acids for peptide purification.^14^ A range of amino acids linked to the 2,7-disulfo-9-fluorenylmethoxycarbonyl (Smoc) group (Scheme 1) is now commercially available. However, their self-assembly properties and biocompatibility have yet to be examined, to the best of our knowledge.

As well as self-assembly properties, Fmoc-amino acids and Fmoc-dipeptides (and mixtures) have good biocompatibility, and hydrogels have a suitable stiffness for cell culture applications. This has been the basis of commercial technology, for example mixtures containing Fmoc-serine (Fmoc-S), Fmoc-diphenylalanine (Fmoc-FF) and/or Fmoc-RGD, marketed by biogelx.^15−17^ Here RGD is a fibronectin-based integrin adhesion motif.^18−20^

Here, we investigate the conformation, self-assembly, and cytocompatibility of three novel Smoc-amino acids (Smoc-aa’s). Conjugates to three amino acids are examined: hydrophobic nonaromatic l-alanine, hydrophobic aromatic l-phenylalanine, and cationic l-arginine (a wide range of other Smoc-aa’s are now available on the market including many analogues of Fmoc-amino acids and those with side chain protecting groups). Depending on pH, Smoc may be charged and hydrophilic, so conjugation to hydrophobic amino acids leads to amphiphilic molecules that may have a self-assembly propensity. Arginine is also able to undergo hydrophobic (π–π stacking) interactions due to the delocalized sp^2^ electrons in the guanidinium group^21^ and so may also aggregate (depending on pH and/or the presence of salt). The conformation of the three Smoc-aa conjugates is probed using circular dichroism (CD) and FTIR spectroscopy. Critical aggregation concentration (CAC) values are determined from fluorescence probe assays, and images of the self-fluorescence are presented, under UV illumination. The CAC values obtained from self-fluorescence are similar to those obtained from thioflavin T (ThT) probe measurements, which are sensitive to β-sheet formation. The presence of such a structure was also evident from the CD and/or FTIR spectra. Self-assembly is investigated using both cryo-TEM imaging and small-angle X-ray scattering (SAXS) which provide information about the morphology and the nanostructure composition and dimensions. Cytocompatibility with L929 fibroblasts is assessed using MTT [3-(4,5-dimethylthiazol-2-yl)-2,5-diphenyltetrazolium bromide] assays, and confocal fluorescence microscopy (laser scanning confocal microscopy, LSCM) imaging is used to show that Smoc-aa’s are located in the cytoplasm. The Smoc-aa’s are shown to have excellent cytocompatibility, with full viability of model L929 fibroblasts (proliferative behavior compared to control) over a range of concentrations below the CAC.

## Experimental Section

### Materials

Smoc-l-Ala (*M*_w_: 471.45 g/mol), Smoc-l-Phe (*M*_w_: 547.55 g/mol), and Smoc-l-Arg (*M*_w_: 556.56 g/mol) were purchased from IRIS Biotech GmbH (Marktredwitz, Germany). Hereafter, for convenience, the l-notation is dropped in the sample names. DMEM and MTT were purchased from Sigma-Aldrich (Gillingham, United Kingdom) and GlutaMAX and penicillin-Streptomycin were purchased from ThermoFisher (Loughborough, United Kingdom). Other materials for cell imaging experiments are listed below.

### Sample Preparation

Samples were dissolved directly in water (native pH) or by addition of NaOH (1.4 wt % solution) for pH 7 solutions. The corresponding pH was measured with a Mettler Toledo FiveEasy pH meter with a Sigma-Aldrich micro pH combination electrode (glass body). Concentration-dependent pH measurements are shown in SI, Figure S1. p*K*_a_ values from group contribution calculation methods were obtained from ChemDraw (version 21.0.0.28).

### UV–Vis Absorption

Spectra were recorded by using a Varian Cary 300 Bio UV–vis spectrometer. Solutions were loaded in a 10 mm light path quartz cell.

### UV–Vis Spectra Modeling

Calculations were performed for Smoc-Ala. The conformation was first energy minimized by using molecular mechanics (MM2, ChemBioUltra 12.0). QM calculations on the generated molecule were then performed using density functional theory (DFT) using Gaussian 16 via Gaussview. The method was time-dependent self-consistent field TD-SCF theory with B3LYP functional and 6-311G basis set, solving for *N* = 6 states with IEFPCM water solvation.^22^

### Fluorescence Spectroscopy

Experiments were carried out by using a Varian Model Cary Eclipse spectrofluorometer. Solutions were loaded in a 10 mm light path Quartz cell. Initially, Thioflavin T (ThT) fluorescence assays were performed in an attempt to identify the critical aggregation concentration (CAC) of the peptides. For the experiments, a series of dilutions in 5.0 × 10^–3^ wt % ThT was prepared starting from a mother solution containing 0.2 wt % peptide in 5.0 × 10^–3^ wt % ThT. The fluorescence of ThT in the dilution series was excited at 440 nm and the emission spectra was measured from 460 to 800 nm. ThT assays were complemented by studying the self-fluorescence of the peptides as a function of the peptide concentration. A series of dilutions were prepared from an initial mother sample containing 0.2 wt % peptide. The solutions were excited at 281 nm, and the emission fluorescence was measured from 300 to 500 nm. The wavelength of excitation was chosen from the corresponding peak of absorption measured in UV–vis experiments.

Additional fluorescence spectra comparing the fluorescence of Smoc-aa’s with that of fluorophores for cell imaging (LSCM) were recorded by using a Hitachi F2500 spectrometer. Smoc-aa’s dissolved in water at a concentration of 0.05 wt % were first gently sonicated and then excited at λ = 405 nm. Spectra were collected from all fluorophores present in the samples for confocal microscopy.

### Circular Dichroism (CD) Spectroscopy

CD spectra were recorded using a Chirascan spectropolarimeter (Applied Photo Physics, Leatherhead, U.K.). Solutions were placed between parallel plates (0.1 or 0.01 mm path length). Spectra were measured with a 0.5 nm step, 0.5 nm bandwidth, and 1 s collection time per step. The CD signal from the water background was subtracted from the CD data of the sample solutions. CD signals were smoothed using the Chirascan Software for data analysis. The residue of the calculation was chosen to oscillate around the average to avoid artifacts in the smoothed curve.

### Fourier Transform Infrared (FTIR) Spectroscopy

Spectra were recorded for samples in D_2_O solutions using a Thermo-Scientific Nicolet iS5 instrument equipped with a DTGS detector, with a Specac Pearl liquid cell with CaF_2_ plates. A total of 128 scans for each sample were recorded over the range of 900–4000 cm^–1^.

### Cryogenic-TEM (Cryo-TEM)

Imaging was carried out using a field emission cryo-electron microscope (JEOL JEM-3200FSC), operating at 200 kV. Images were taken in bright field mode and using zero loss energy filtering (omega type) with a slit width of 20 eV. Micrographs were recorded using a Gatan Ultrascan 4000 CCD camera. The specimen temperature was maintained at −187 °C during the imaging. Vitrified specimens were prepared using an automated FEI Vitrobot device using Quantifoil 3.5/1 holey carbon copper grids with a hole size of 3.5 μm. Just prior to use, grids were plasma cleaned using a Gatan Solarus 9500 plasma cleaner and then transferred into the environmental chamber of a FEI Vitrobot at room temperature and 100% humidity. Thereafter 3 μL of sample solution was applied on the grid and was blotted twice for 5 s and then vitrified in a 1/1 mixture of liquid ethane and propane at temperature of −180 °C. The grids with vitrified sample solution were maintained at liquid nitrogen temperature and then cryo-transferred to the microscope.

### Small-Angle X-ray Scattering (SAXS)

Synchrotron SAXS experiments on solutions were performed using a BioSAXS setup on BM29 at the ESRF (Grenoble, France).^23,24^ A few microliters of samples were injected via an automated sample exchanger at a slow and reproducible rate into a quartz capillary (1.8 mm internal diameter) in the X-ray beam. The quartz capillary was enclosed in a vacuum chamber to avoid parasitic scattering. After the sample was injected into the capillary and reached the X-ray beam, the flow was stopped during the SAXS data acquisition. The *q* range was 0.005–0.48 Å^–1^, with λ = 1.03 Å. and the images were obtained using a Pilatus3–2 M detector. Data processing (background subtraction and radial averaging) was performed using dedicated beamline software ISPYB.

### MTT Assays

L929 murine fibroblasts were maintained with DMEM medium supplemented with 10% fetal bovine serum (FBS), 2 mM of GlutaMAX and penicillin-streptomycin. The cells were incubated at 37 °C with a controlled atmosphere of 5% CO_2_ inside a cell incubator. A period 24 h prior to the assay, L929 cells were detached with trypsin and transferred to 96-well plates at a density of 6 × 10^3^ cells per well. Cells were then incubated with Smoc-aa’s dissolved in the medium at concentrations of 0.1 to 2 × 10^–4^ wt % for 72 h inside the cell incubator. Images of the cells incubated with the Smoc amino acids were obtained before adding the MTT by standard phase contrast microscopy. After the incubation, the wells were washed 3 times with PBS and incubated for 4 h at 37 °C with 100 μL of 0.05 wt % MTT dissolved in DMEM without phenol red. The resulting formazan crystals were dissolved by adding 100 μL of DMSO and incubating the plate at 37 °C for 45 min protected from the light with aluminum foil. Absorbance values were determined at 560 nm using an automatic plate reader. Cell survival was expressed as a percentage of viable cells in the presence of Smoc-aa’s, compared to control cells grown only in DMEM without any Smoc-aa’s.

### Statistical Analysis

For the cytocompatibility data, absorbance at 560 nm was measured for all samples, and the background removed. Samples incubated with the Smoc-aa’s were compared using the control as standards to calculate the percentage of living cells when compared to control. Data are presented as mean ± SD using a bar chart. The analysis used was the ordinary one-way ANOVA to determine any statistically significant difference using the F distribution between different groups (*n* = 3). The parameter for evaluating the variance of the samples was the Brown-Forsythe test for the analysis of variance. Since we had many groups in this sample, a Bonferroni correction was applied to protect from type I error (false positives) and probability *p* < 0.05 was regarded as statistically significant. The software used for the analysis was GraphPad Prism 8.

### Cell Culture and Laser Scanning Confocal Microscopy (LCSM)

L929 cells were cultured in Dulbecco’s modified Eagle medium (DMEM), with 10% FBS and 2 mM of glutamine (Thermo Fisher Scientific, Massachusetts) and incubated in a controlled atmosphere with 5% CO_2_ at 37 °C. Glass coverslips were placed into 24 well plates and 5 × 10^4^ cells were added and left to proliferate for 24 h to reach confluence. The day after, coverslips were washed three times with PBS to remove excess serum and cell debris. Smoc-aa solutions were prepared at 0.05 wt % in DMEM and added into the 24 well plate. The samples were prepared along a control consisting only of DMEM and incubated for 72 h at 37 °C. After incubation, samples were washed 3 times with PBS to remove unadhered cells and any remaining Smoc-aa’s. To stain acidic organelles (e.g., endosomes), samples were incubated with Lysotracker Deep Red (Invitrogen, California) in culture media for 2 h and calcein green AM for 15 min inside the cell incubator. After further washing with PBS, fixation was performed with 4% paraformaldehyde, and phalloidin-Texas red (Invitrogen, California) was used to stain actin filaments. For this, fixed cells were washed three times with PBS, incubated with Triton X-100 0.1% for 15 min at room temperature, washed again, and then incubated with a solution containing 6 μM of phalloidin Texas red at room temperature for 2 h. After this period, cells were washed again three times with PBS, mounted on glass slides with antiquenching mounting media Fluoromount-G (Thermo Fisher Scientific, Massachusetts). Imaging was carried out with a confocal microscope (Leica TCS SP8, Mannheim, Germany) using appropriate laser sources to match the excitation wavelengths for each fluorophore. An argon ion laser with excitation at λ = 405 nm was used. The controls were treated with the same fluorophores and fluorescence conditions used for the samples incubated with the Smoc-aa’s. Image treatment was performed using ImageJ-Fiji software.^25^

### In Cellulo Fluorescence Imaging

The fluorescence of Smoc-amino acids in living cells was observed by cultivating L929 fibroblasts in 24 well plates until they reached confluence, incubating with 0.05 wt % of each Smoc-aa for 4 h, washing with PBS three times, and detaching with trypsin. After removing the trypsin by centrifugation, the cells were counted and fixed with PFA 4%, washed and fluorescence measurements were performed using a Hitachi F2500 fluorimeter with excitation at λ = 281 nm with each sample containing 5 × 10^4^ cells. The resulting graphs were exported and analyzed using the software Origin.

### AFM Imaging of Cell Surfaces

For AFM, 5 × 10^4^ L929 cells were seeded in 24 well plates containing mica sheets at the bottom 1 day before incubation. Then, cells were washed and incubated with 0.5 wt % of each Smoc-aa for 72 h in DMEM at 37 °C inside a CO_2_ cell incubator. Prior to fixation, cells were washed three times and fixed with cold ethanol for 10 min to ensure deposition and fixation on the mica sheets. After washing the ethanol with distilled water, areas from 5 × 5 μm up to 40 × 40 μm of the mica sheets containing the cells were imaged using a Park NX10 (Park Systems, South Korea) atomic force microscope at LNNANO (CNPEM, Campinas, Brazil) in tapping mode with the cantilever operating around 240 kHz (SA). The resulting topographical images were analyzed with the software Gwyddion.

## Results

The self-assembly of the three Smoc-amino acid (Smoc-aa) conjugates (Scheme 1) representing conjugates with aliphatic, aromatic, or charged (cationic) amino acids was first examined. These studies were conducted at native pH (pH 3.7–4, SI, Figure S1) and, relevant to biological applications, at pH 7. The native pH solutions are close to the calculated p*K*_a_ values (p*K*_a_ = 3.74 for Smoc-Ala, p*K*_a_ = 3.52 for Smoc-Phe, p*K*_a_ = 3.55 for Smoc-Arg), while pH 7 is significantly higher. The studied pH values are well below the p*K*_a_ of arginine (p*K*_a_ 12.5), and so arginine will be fully protonated under the conditions studied.

**Figure 1:**
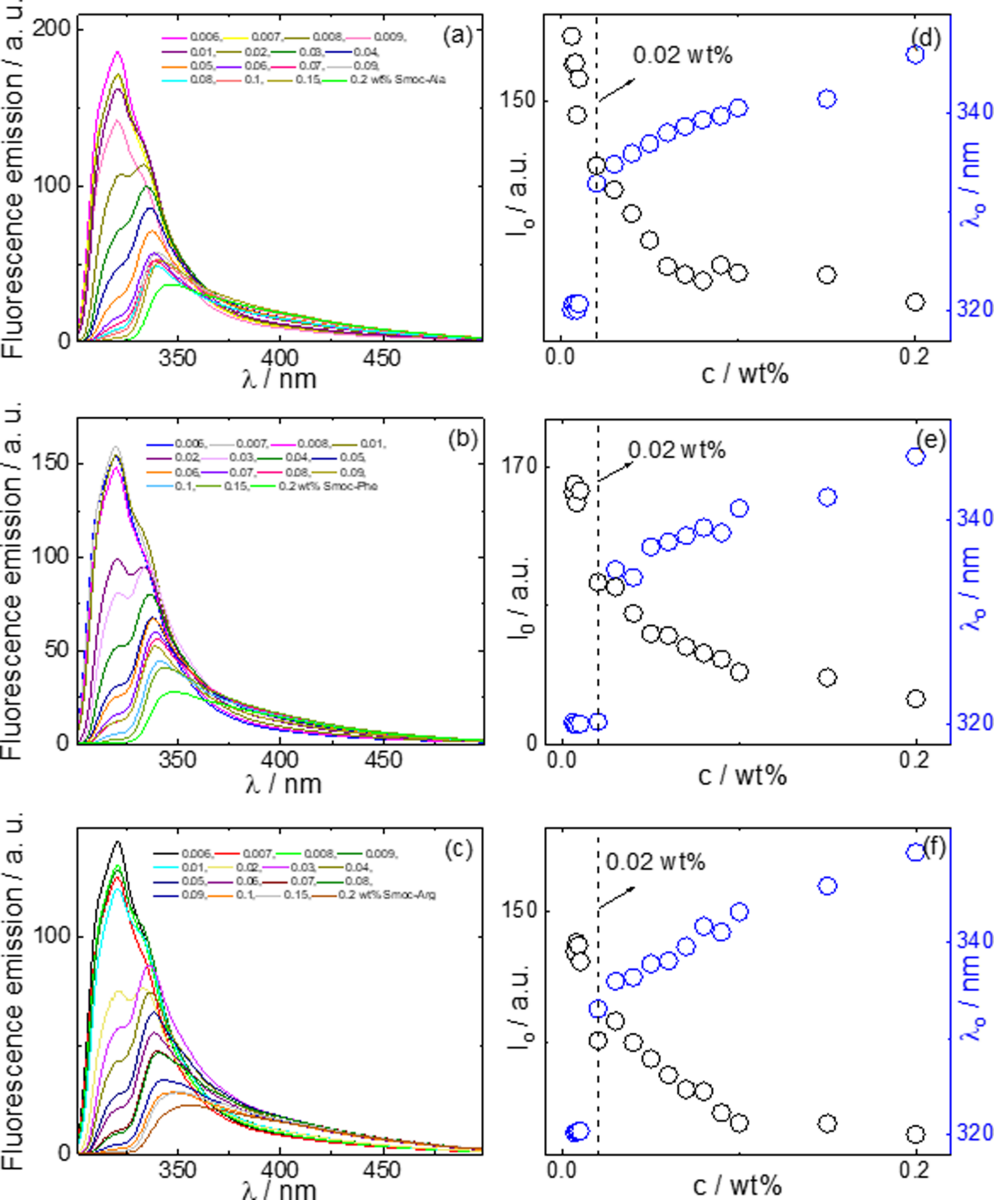
(a–c) Self-fluorescence emission spectra (native pH) at the concentrations shown along with (d–f) intensity (*I*_0_) and position (λ_0_) of the maximum in the self-fluorescence emission spectra: (a, d) Smoc-Ala, (b, e) Smoc-Phe, and (c, f) Smoc-Arg.

It was found to be possible to determine critical aggregation concentration (CAC) values for the three Smoc-aa conjugates using the fluorescence of the Smoc group. Figure 1 shows fluorescence spectra at native pH as a function of concentration along with plots of the peak intensity and position. For all three samples, there is a sharp change in the spectra starting at 0.02 wt % (identified as the critical aggregation concentration, CAC), with the development of a sharp fluorescence peak red-shifted with respect to the peak observed at low concentration at 319–320 nm, associated with unaggregated molecules. This is further red-shifted as concentration increases up to 0.1 wt %, at which concentration for all three samples, the original “monomer” peak in the spectrum has disappeared. The observed red shift in the fluorescence emission spectra suggests the formation of J-aggregates with head-to-tail arrangement of the molecules,^26,27^ here containing Smoc fluorophores, above the CAC. Similar results were obtained at pH 7, the same CAC value 0.02 wt % being found for all three Smoc-aa’s (SI, Figure S2).

As a second test of CAC, we also performed assays using thioflavin T (ThT), a fluorescent dye that binds β-sheet amyloid structures.^28−30^ The corresponding spectra are shown in SI, Figure S3a for native pH, along with plots of the concentration dependence of the peak intensity in SI, Figure S3b. These reveal CAC values (0.02 ± 0.005) wt % (from discontinuities in λ_0_, SI, Figure S3a, with slightly different estimates based on *I*_0_, SI, Figure S3b) for all three Smoc-aa’s, which is in excellent agreement with the values obtained from the self-fluorescence of Smoc (Figure 1). At pH 7, the CAC values for all three Smoc-aa’s (0.02–0.04 wt %, SI, Figures S4 and S5) are also in agreement with those obtained from Smoc self-fluorescence. The existence of apparent CACs in ThT assays suggests that aggregation into β-sheet structures may occur above the CAC for all three Smoc-aa’s. The secondary structures were then further probed by using CD (circular dichroism) and FTIR spectroscopy.

**Figure 2:**
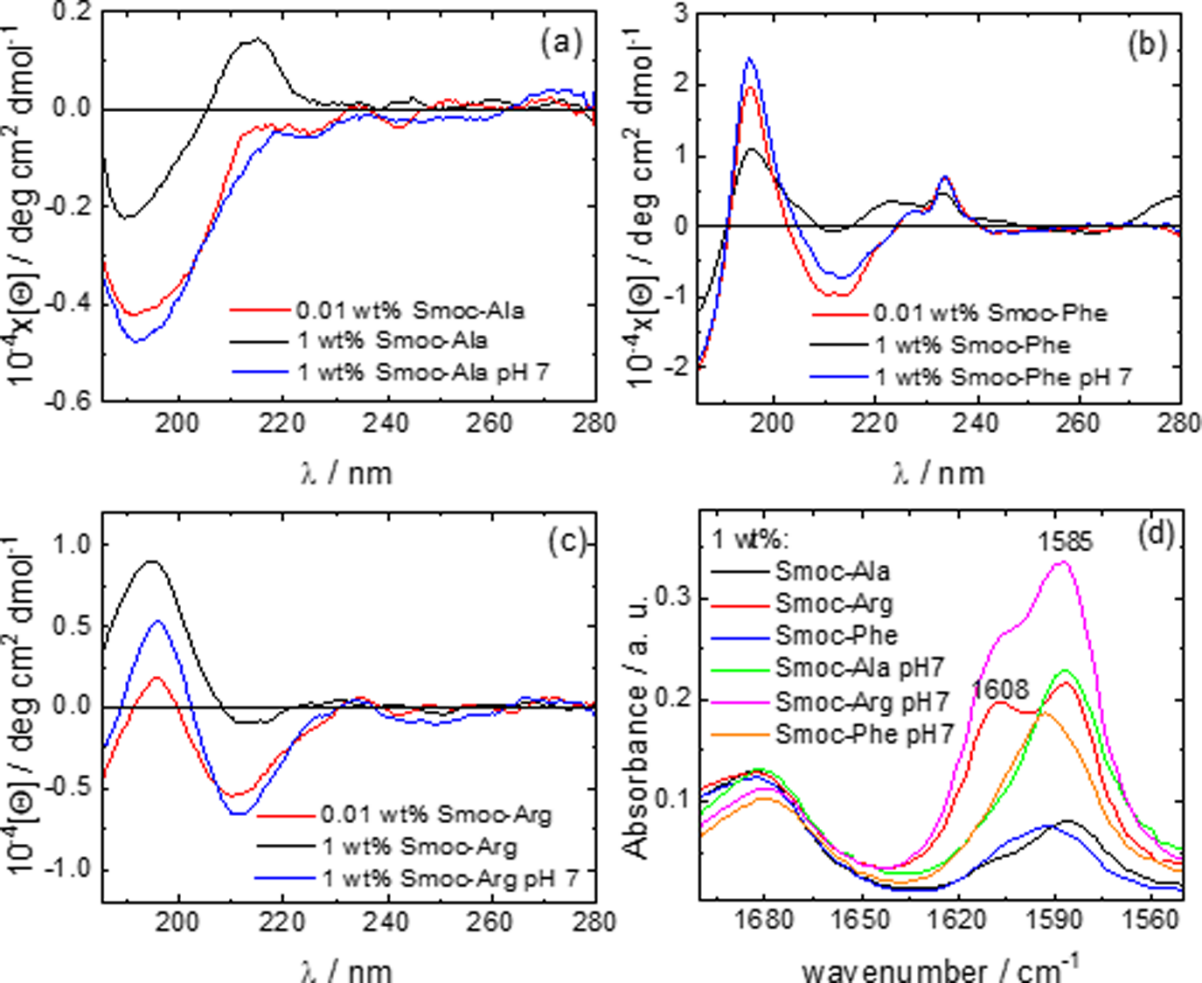
(a–c) CD and (d) FTIR spectra for the Smoc-aa’s at native pH and pH 7; CD data measured at concentrations below and above the CAC.

CD spectra measured below and above the CAC are presented in Figure 2a–c and FTIR spectra (measured above the CAC) are shown in Figure 2d. The CD spectra for Smoc-Ala (Figure 2a) show broad negative minima in the range 190–200 nm, and above the CAC (1 wt %), at pH 4, a broad positive maximum near 215 nm, although this is absent at pH 7. These results suggest a predominantly disordered structure,^31−33^ although FTIR (to be discussed shortly) indicates the presence of some β-sheet structure above the CAC. Below the CAC (0.01 wt %), the CD spectrum has a stronger negative minimum centered at 192 nm, with no positive maximum. These features indicate a disordered conformation below the CAC.^31−33^ The CD spectra measured for Smoc-Phe and Smoc-Arg samples show characteristic features associated with predominant β-sheet structure,^31,32,34^ namely, positive maxima near 200 nm and negative minima at 216 nm. A sharp peak near 233 nm is present in the spectra for Smoc-Phe and may reflect a contribution from Phe chromophores as well as Smoc; the latter leads to a peak at this position in the spectra for Smoc-Arg and, to some extent, Smoc-Ala. The UV absorption spectra measured (SI, Figure S6) indeed show fine features at this wavelength. Quantum mechanical calculations using density functional theory (DFT) are able to semiquantitatively reproduce these features (taking Smoc-Ala as an example) as exemplified by the band positions and relative absorbance (SI, Figure S6). The CD spectra show very similar features at native pH 4 and 7.

Considering the FTIR spectra in Figure 2d, the peak at 1683 cm^–1^ for all three samples is in the range typically ascribed to modes associated with the antiparallel β-sheet secondary structure.^30,35^ However, the data shows that it is present for Smoc-Ala (which CD shows does not form β-sheets) and we therefore assign this mode to carbamate modes, as reported for Fmoc-dipeptides around 1685–1690 cm^–1^.^36,37^ For Smoc-Arg the peaks at 1606 and 1587 cm^–1^ are due to arginine guanidinium group modes,^38−41^ although the former also overlap with the characteristic β-sheet peak.^30,35^ Similarly, the peak at 1604 cm^–1^ for Smoc-Phe can be assigned to β-sheet formation (since there are no Phe side chain peaks at this position), and there is also a shoulder peak at this position for Smoc-Ala. The peak at 1585 cm^–1^ for Smoc-Ala is due to a fraction of unaggregated molecules with free carboxyl groups,^42^ consistent with the observed CD spectra (Figure 2a).

In summary, the CD spectra indicate β-sheet conformation for Smoc-Phe and Smoc-Arg whereas Smoc-Ala predominantly adopts a disordered conformation. The Smoc-aa conformations already adopt these conformations below the CAC, but the aggregation is associated with the formation of extensive β-sheet structures for Smoc-Phe and Smoc-Arg but not for Smoc-Ala.

**Figure 3:**
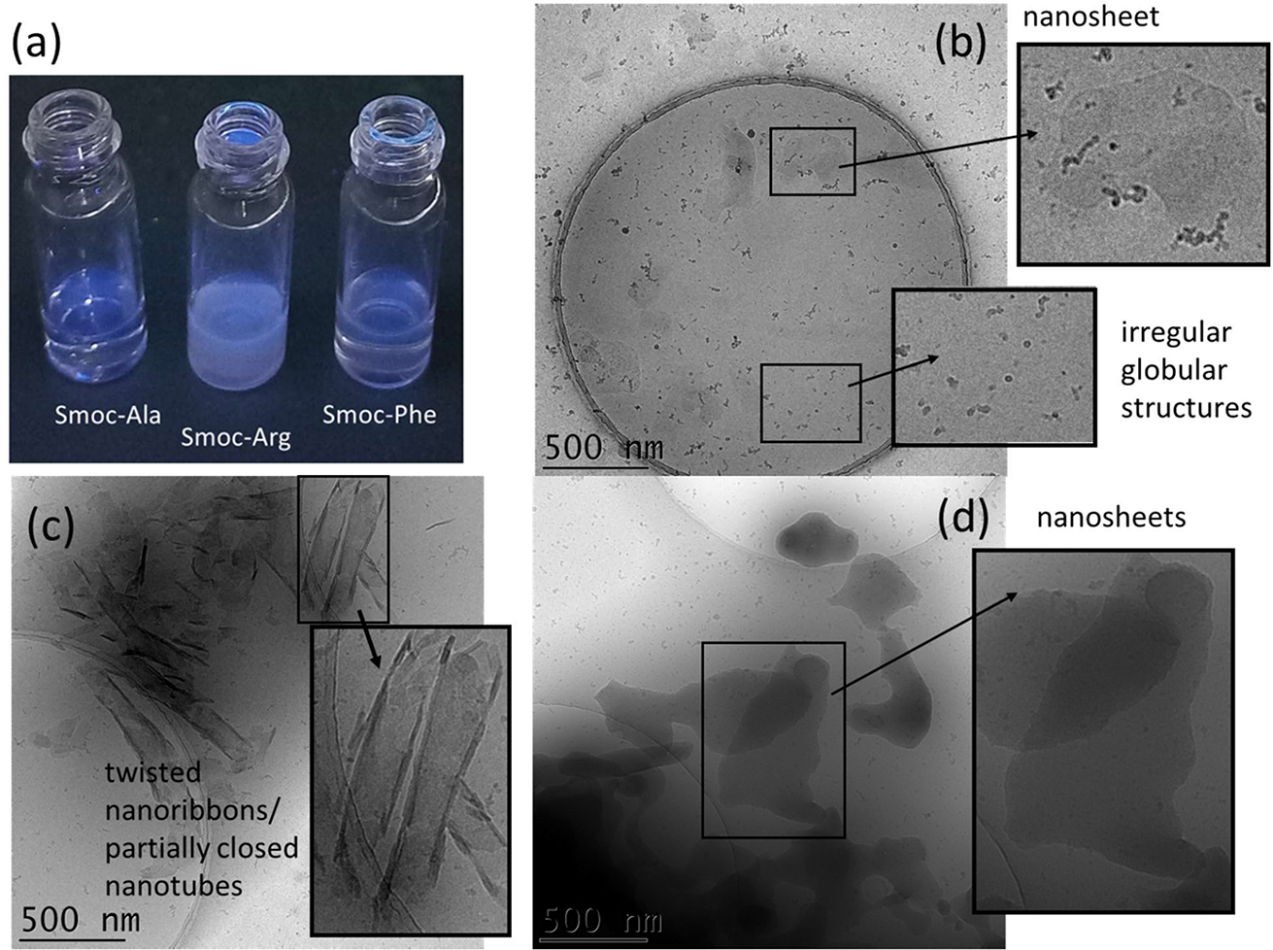
(a) Fluorescence images obtained for 1 wt % samples with λ = 254 nm UV lamp. Cryo-TEM images (native pH 4) with selected enlarged nanostructures: (b) 1 wt % Smoc-Ala, (c) 1 wt % Smoc-Phe, (d) 0.5 wt % Smoc-Arg.

The Smoc-aa’s exhibit fluorescence when illuminated under UV light (λ = 254 nm).^13^Figure 3a shows images for 1 wt % samples, all of which exhibit pronounced blue fluorescence. Images for samples at a concentration below the CAC in SI, Figure S7, also show blue fluorescence for all three samples, although with lower intensity.

The self-assembly behavior of the three Smoc-aa’s was investigated using both cryo-TEM imaging and SAXS, which together provide comprehensive information on the type of nanostructure. Selected cryo-TEM images at native pH are shown in Figure 3b–d, and additional images are provided in SI, Figure S8. Smoc-Ala forms sparse nanosheets coexisting with irregular globular structures, as is evident from the micrograph in Figure 3b. In contrast, Smoc-Phe remarkably self-assembled into twisted nanoribbons along with some closed nanotubes (Figure 3c). Smoc-Arg shows extensive nanosheet structures across the TEM grid (Figure 3d), with strong contrast suggesting that they are thicker than those formed by Smoc-Ala. As with the spectroscopic studies, cryo-TEM was also performed on samples at pH 7 and selected images are shown in SI, Figure S9. The results show that Smoc-Ala shows only a few globular aggregates, Smoc-Arg forms nanosheets as at native pH and Smoc-Phe forms extended nanostructures, here fibrils (deposited on the TEM grid mesh).

**Figure 4:**
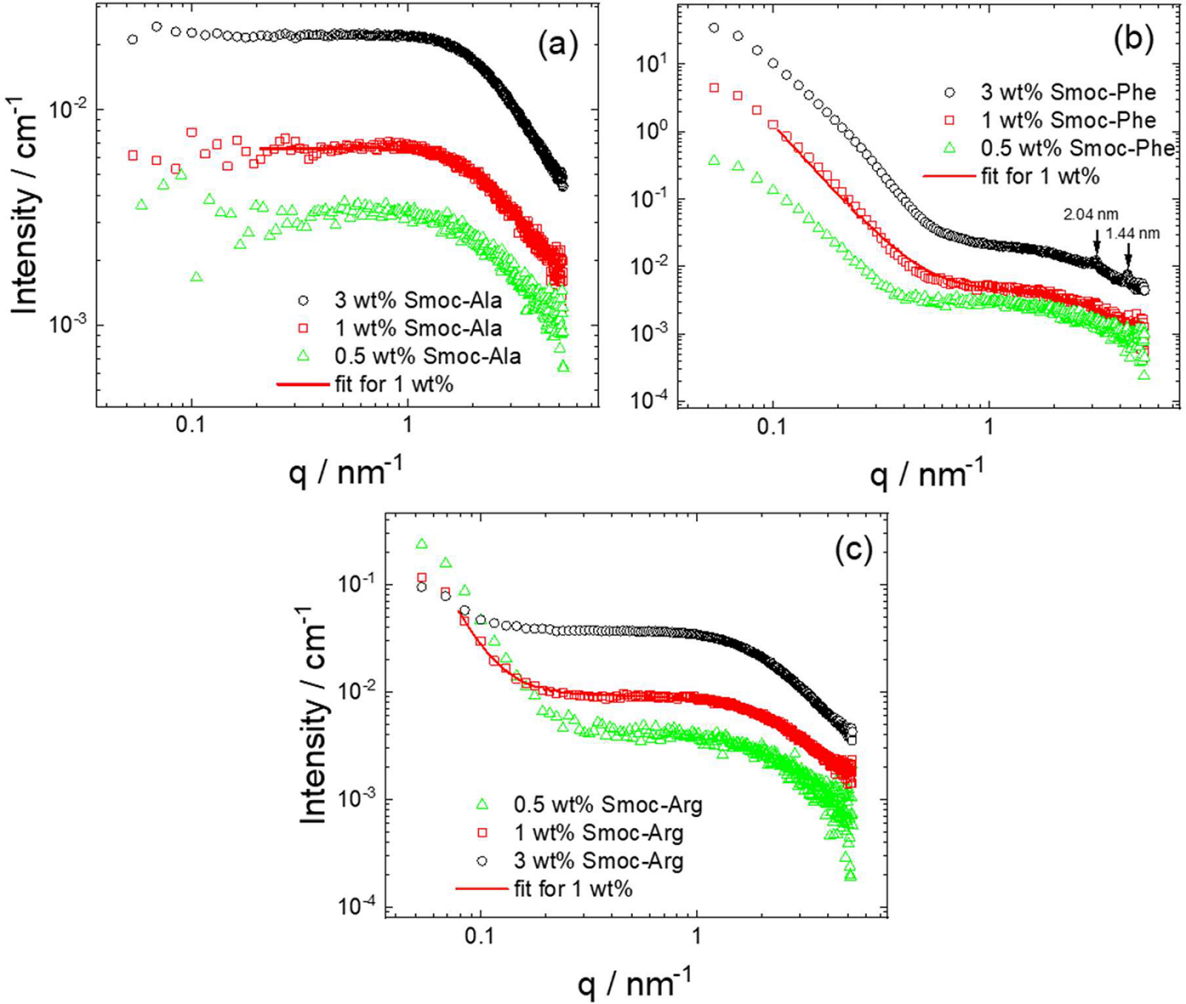
SAXS data at the concentrations shown: (a) Smoc-Ala, (b) Smoc-Phe (Bragg peaks indicated), and (c) Smoc-Arg. For ease of visualization, only every 3rd data point is shown.

Real-space imaging through cryo-TEM was complemented by small-angle X-ray scattering (SAXS) which provides the form factor of self-assembled structures^43^ in solution at concentrations above the CAC. Figure 4 shows the measured data (at native pH) along with form factor fits (fit parameters are listed in SI, Table S1). The SAXS data for Smoc-Ala (Figure 4a) show that aqueous solutions of this molecule mainly comprise monomers. This is consistent with the spectroscopic results, although cryo-TEM does indicate the presence of a few self-assembled nanosheet structures (Figure 3c). The data for Smoc-Phe (Figure 4b) in contrast show a low *q* intensity upturn, indicating self-assembly, along with a plateau at high *q* arising from coexisting monomers. Similar features are observed in the SAXS data at pH 7, as is evident from SI, Figure S10, with monomer features at high *q* and upturns in the intensity at low *q*, indicative of aggregated species (more noticeable at pH 7 for Smoc-Ala compared to native pH). At the highest concentration studied (3 wt %), Bragg peaks are observed for Smoc-Phe at wavenumber *q* = 3.08 nm^–1^ (*d* = 2.04 nm) and *q* = 4.34 nm^–1^ (*d* = 1.44 nm) (Figure 4b), which are associated with the high degree of order within the twisted nanosheet/nanotube structures formed by this peptide.

Because self-assembly is observed for the Smoc conjugates in aqueous solution and since related Fmoc-peptides and mixtures with Fmoc-amino acids can form hydrogels,^15−17,44,45^ we examined potential hydrogelation (including variation of pH up to pH 12 and with the presence of organic cosolvents), however, none was observed for samples containing up to 3 wt % Smoc-aa.

**Figure 5:**
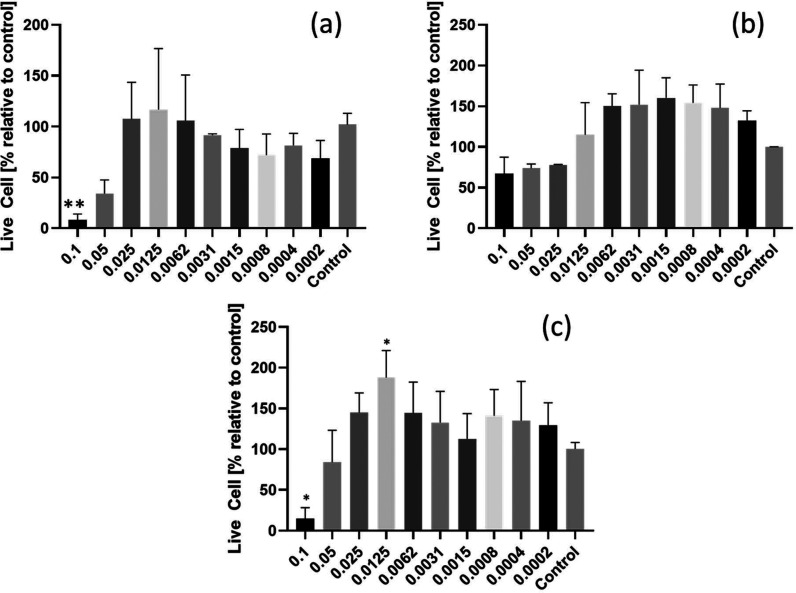
Cytocompatibility from MTT assays after 72 h: (a) Smoc-Ala, (b) Smoc-Phe, and (c) Smoc-Arg. Concentration of the Smoc amino acids is expressed in wt %: *p below 0.05, **p value below 0.01.

Inspired by the utility of Fmoc conjugates (to peptides or amino acids) in cell culture biomaterials, we performed initial cytocompatibility studies with the novel Smoc-aa’s using L929 fibroblasts with MTT assays. The results are shown in Figure 5. In general, the three Smoc-aa’s are well tolerated at concentrations below the CAC, although there is notable cytotoxicity at the highest two concentrations examined (0.05 and 0.1 wt %) above the CAC. This is also evident in the optical microscopy images in SI, Figures S11−S13, which show significant numbers of rounded cells at 0.1 wt % in contrast to the dense arrays of cells presenting protrusions observed for all three samples at 0.0625 wt %, consistent with attachment and a healthy morphology. For Smoc-Phe and Smoc-Arg there is actually a proliferative effect at lower concentrations, with cell viabilities >100% with respect to control. Smoc-Ala shows lower viability than Smoc-Phe or Smoc-Arg, although not significantly different from the control. This may reflect the lower aggregation propensity of Smoc-Ala, as discussed above.

The uptake and partitioning of the Smoc-aa conjugates in cells were examined using LCSM imaging with L929 fibroblasts. Imaging was possible since a partial emission of the Smoc group was observed when excited at λ = 405 nm, a common wavelength that is accessible in most confocal microscopes for the excitation of the DNA stain 4′,6-diamidino-2-phenylindole (DAPI).^46^ Fluorescence spectra of the three Smoc-aa’s and the other fluorophores applied to image cell components are presented in SI, Figure S14.

**Figure 6:**
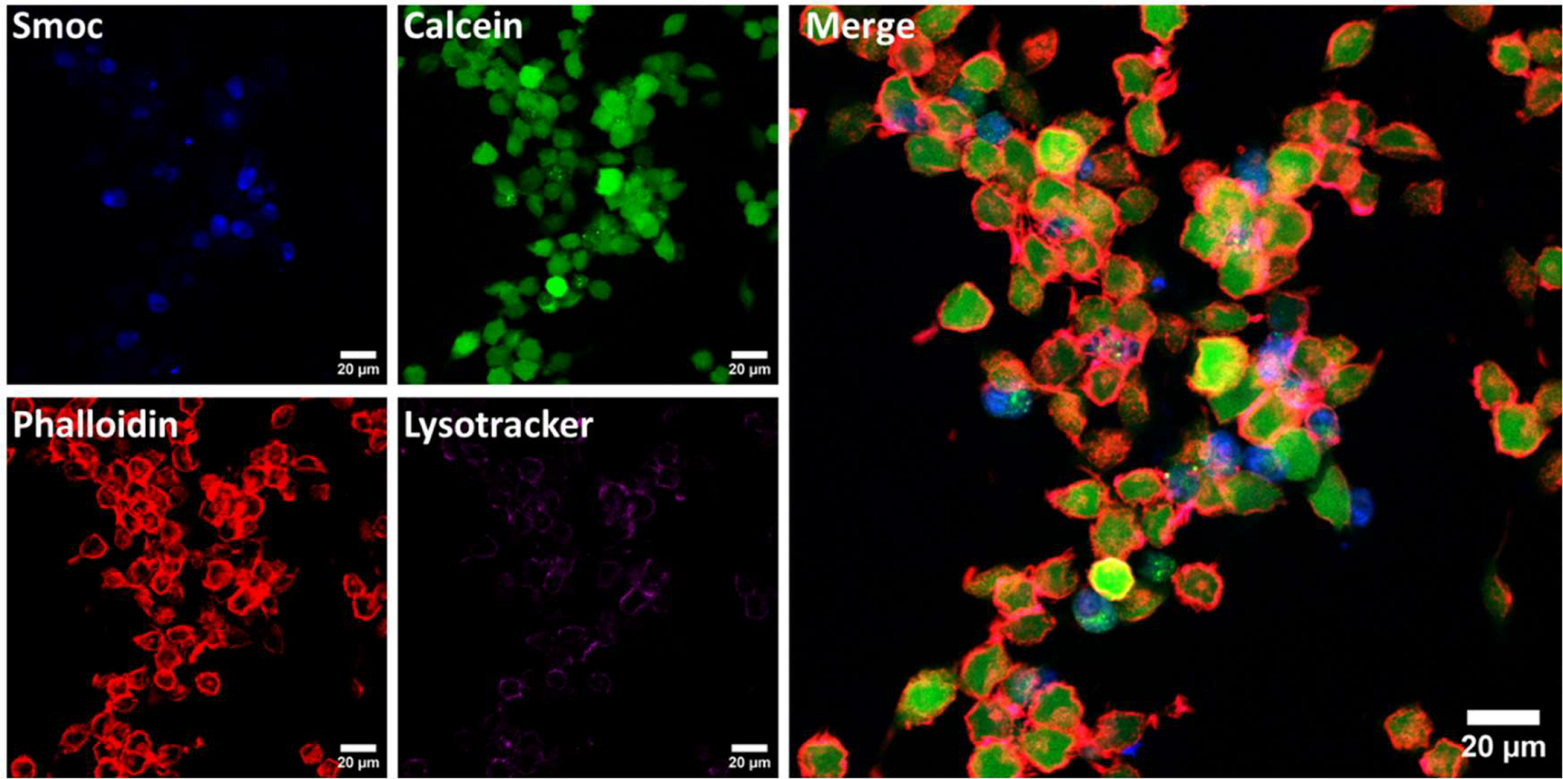
Confocal microscopy images of L929 cells incubated with 0.05 wt % Smoc-Arg in DMEM for 72 h. The blue channel shows Smoc fluorescence, the green channel shows calcein fluorescence, the cytoskeleton stained with phalloidin Texas red is represented in red, and the endosomal system is colored in magenta by Lysotracker Deep Red. The last panel is a merged image of all four channels.

LSCM images in Figure 6 show that some L929 cells incubated with Smoc-Arg were stained and showed fluorescence when excited at λ = 405 nm. The colocalization with the cytoplasm stained with calcein green and the F-actin cytoskeleton stained by phalloidin conjugated with Texas red indicates cell viability and that Smoc-Arg is mainly located in the cytoplasm, not the nucleus.^47,48^ The lack of co-localization with acid organelles, especially endosomes and lysosomes marked by the probe Lysotracker Deep Red, also indicates that the Smoc-aa was not trapped inside the endosomal system, a common destiny for exogenous molecules internalized by cells, providing additional evidence for uptake of the amino acids into the cytoplasm and/or cell membrane.^49−51^

Similar results were obtained for Smoc-Ala (SI, Figure S15) and Smoc-Phe (SI, Figure S16). The control incubated with only DMEM for 72 h in the absence of the Smoc-aa’s did not present any relevant fluorescence in the blue channel when excited under the same conditions as the samples incubated with the Smoc-amino acids (SI, Figure S17). Thus, LSCM shows that all Smoc-aa’s are not trapped in endosomes, but are able to internalize within the cytoplasm or at least anchor in the cell membrane, as observed in the *z* series image stack obtained for internalized fibroblast cells presenting Smoc fluorescence in multiple focal planes (SI, Video S1).

We examined if the intrinsic Smoc fluorescence can be used to determine whether the peptides are present as monomers or aggregates in cells. Fluorescence spectra were measured for L929 fibroblasts incubated with the Smoc-aa’s. The results are shown in SI, Figure S18. The peak maximum at 333–334 nm corresponds to that of the control, which is ascribed to the presence of tryptophan (which has a fluorescence peak maximum at this position) in the cells and media. Thus, the presence of Smoc-aa aggregates in the cells cannot clearly be concluded, however it can be noted that no peak at 320 nm corresponding to Smoc-aa monomers is observed (such a peak is observed for Fmoc-Arg^52^). It was also noted that at a concentration 0.05 wt % (where cytotoxicity is observed, Figure 5), the Smoc-aa’s interact with cell surfaces, changing the topography as probed by AFM. Images are shown in SI, Figure S19, and the R.M.S. roughness was substantially reduced from 27.8 nm for untreated L929 cells to 12.7 nm for L929 cells exposed to Smoc-Ala, 14.9 nm for L929 cells exposed to Smoc-Phe and 9.1 nm for L929 cells exposed to Smoc-Arg. Thus, all three Smoc-aa’s interact with cell membranes and significantly reduce roughness.

## Discussion and Conclusions

The combination of cryo-TEM and SAXS provides unique insight into the self-assembly of the three Smoc-aa’s. SAXS reveals significant fractions of unaggregated peptide for both Smoc-Ala and Smoc-Arg, although Smoc-Phe seems to be fully self-assembled above the CAC. Very little difference is observed in the self-assembly behavior at native pH 4 and 7. Cryo-TEM directly shows the morphology of the population of self-assembled structures present. The distinct nanostructures formed by the three Smoc conjugates are remarkable. Smoc-Ala forms sparse small nanosheets coexisting with small globular aggregates, while Smoc-Arg self-assembles into extended nanosheet structures. In complete contrast, twisted nanosheet and nanotube structures are observed for Smoc-Phe. It is notable that fibrillar structures, modeled with a hollow nanotube structure were previously reported for Fmoc-PhePhe.^53^ The self-assembly behavior of Smoc-Phe, in contrast to the two conjugates bearing nonaromatic residues, presumably results from enhanced π–π stacking interactions as observed previously for Fmoc-Phe,^54−57^ Fmoc-Tyr,^58^ etc. A possible explanation for the nanosheet formation by Smoc-Arg is that it results from the special properties of the arginine guanidinium group, such as its ability to undergo cation-π interactions with aromatic groups. This has been modeled in detail^59,60^ and it has been shown that due to the *sp*^2^ nature of the delocalized amine bonds in the guanidinium group, arginine can participate in hydrophobic π–π interactions (Arg–Arg or Arg–X, where X is a hydrophobic aromatic residue).

It should be noted that only selected Smoc-aa’s have been investigated in this initial report, which are representative of different classes of amino acids. A full range of Smocamino acids (including those with protecting groups), i.e., a range of analogues of Fmoc-amino acids, is now available commercially and can be used to prepare Smoc-peptides. The self-assembly properties and their biocompatibility and applications are promising subjects for further investigation.

The self-fluorescence of the Smoc moiety enables the measurement of CAC values without the need for added fluorescent dye probes, i.e., without any potential perturbation of aggregated structures. The values obtained are the same as those obtained from measurements using the ThT probe of β-sheet formation, indicating the presence of β-sheet structure above the CAC. In addition, the fluorescence under UV illumination was used in LCSM imaging since fluorescence was observed at a wavelength available with argon ion laser sources for confocal microscopy. The imaging revealed uptake of the Smoc-aa peptides into the cell cytoplasm and little or no entrapment inside the endosomal system. In the future, this technique could be applied to Smoc-attached cell targeting peptides, which may localize in specific organelles, a subject of great interest for further research.

None of the Smoc-aa’s evaluated presented any statistically significant decrease in cell survivability at concentrations of 0.05 wt % and below when compared with control groups, indicating that they are well tolerated by L929 cells. The cytocompatibility at concentrations below the CAC is excellent, especially for Smoc-Phe and Smoc-Arg, which show viabilities >100% compared to control. The cytotoxicity at high concentration appears to be correlated to the formation of self-assembled nanostructures. AFM shows an interaction between the Smoc-aa’s and cell membranes, in addition to their presence within the cell shown by LSCM. Future work should examine the origins of the cytotoxicity, which might be due for example simply to a localized high density presentation of bioactive motifs.^61−64^

## Supporting Information Available

Concentration–pH measurements, measured Smoc fluorescence spectra, ThT fluorescence assays of CAC data and corresponding spectra, measured and calculated UV–vis absorption spectra, fluorescence images obtained below CAC, additional cryo-TEM images at pH 4 and pH 7, SAXS data at pH 7, optical microscopy images of cells, confocal microscopy images of L929 fibroblasts and associated fluorescence spectra, AFM images of cell surfaces, and table of SAXS fit parameters (PDF)
Video S1: The *z* series image stack obtained for internalized fibroblast cells presenting Smoc fluorescence in multiple focal planes (AVI)

## Supplementary Material

bm3c00860_si_001.pdf

bm3c00860_si_002.avi
