# Supplementary material for: Self-Assembly and Cytocompatibility of Amino Acid Conjugates Containing a Novel Water-Soluble Aromatic Protecting Group
Source: Biomacromolecules. 2023 Nov 1;24(11):5403–13. doi: 10.1021/acs.biomac.3c00860 (PMC10646988; doi:10.1021/acs.biomac.3c00860)
Supplement: Supplementary file 1 — bm3c00860_si_001.pdf [file bm3c00860_si_001.pdf]

Supplementary information

Self-Assembly and Cytocompatibility of Amino Acid Conjugates Containing a Novel Water-Soluble Aromatic Protecting Group

Valeria Castelletto,[1] Lucas de Mello,[1,2] Emerson Rodrigo da Silva,[2] Jani Seitsonen,[3] Ian W Hamley[1,*]

[1] School of Chemistry, Food Biosciences and Pharmacy, University of Reading, Whiteknights, Reading RG6 6AD, U.K.

[2] Departamento de Biofísica, Universidade Federal de São Paulo, São Paulo 04023-062, Brazil

[3] Nanomicroscopy Center, Aalto University, Puumiehenkuja 2, FIN-02150 Espoo, Finland

* Author for correspondence: I.W.Hamley@reading.ac.uk

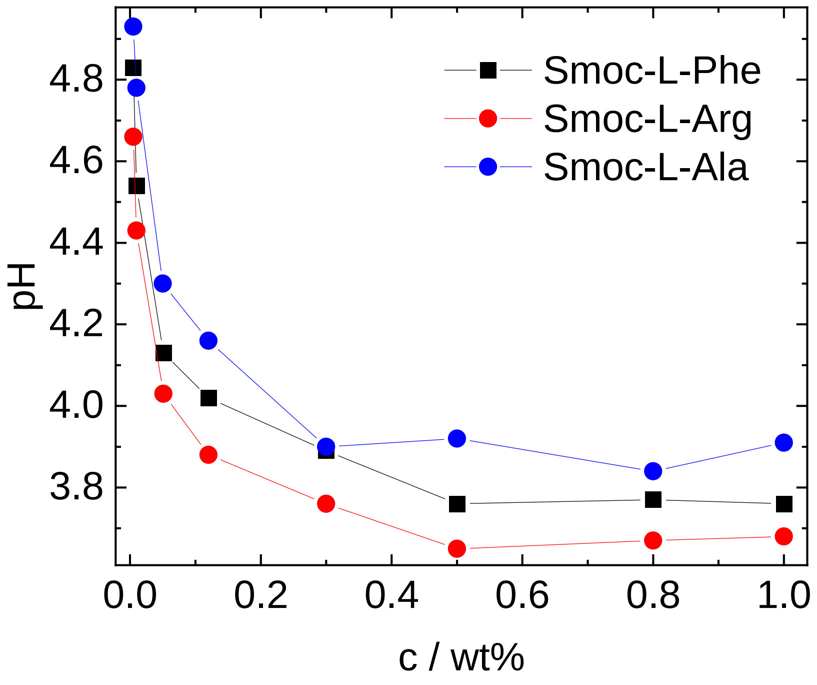

Figure S1. pH dependence on concentration for the three Smoc-amino acids.

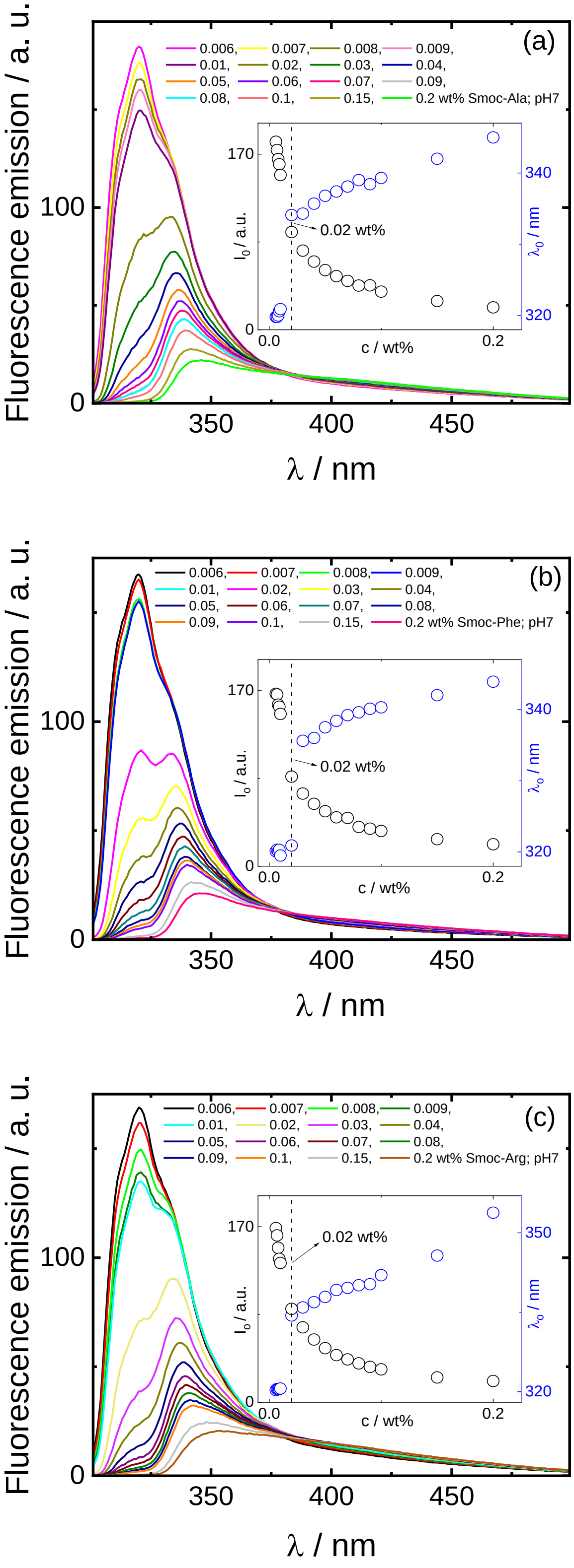

Figure S2. Self-fluorescence emission spectra at the concentrations shown, at pH 7, along with (insets) intensity (I_0) and position (λ_0) of the maximum in the self-fluorescence emission spectra. (a) Smoc-Ala, (b) Smoc-Phe, (c) Smoc-Arg.

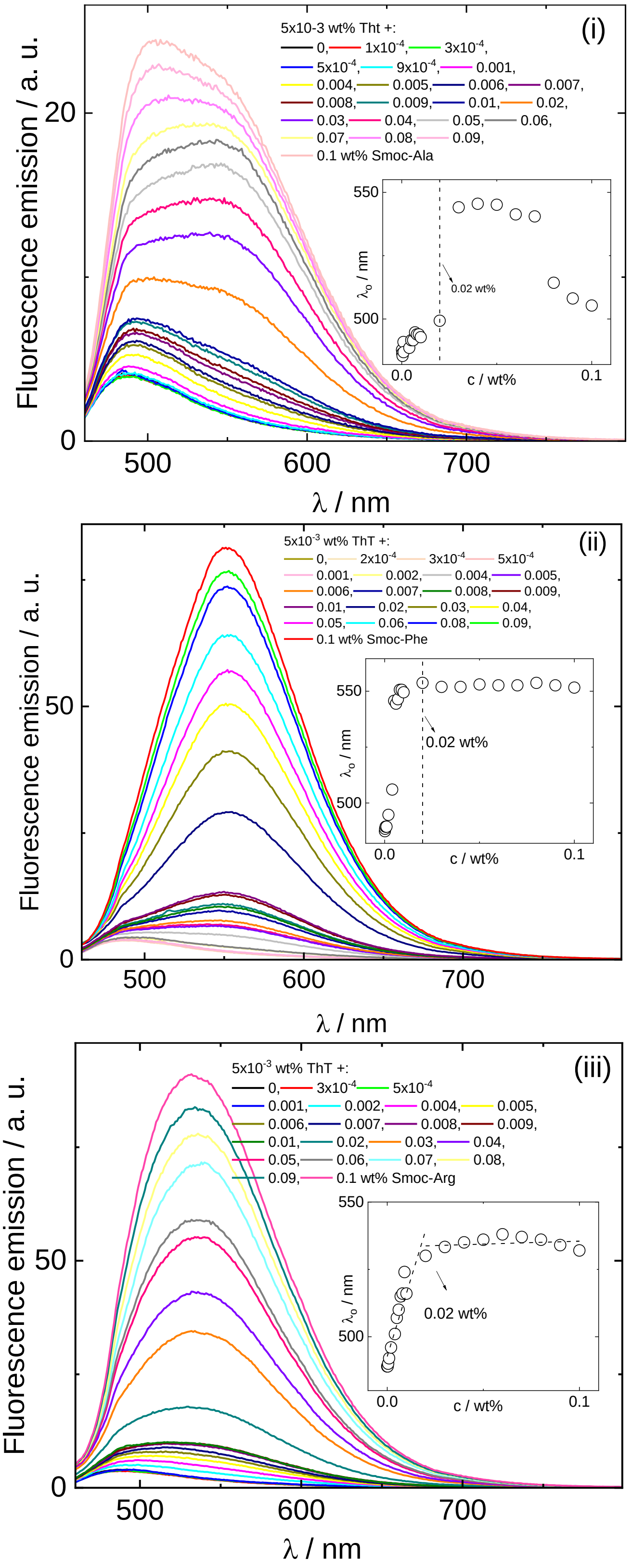

Figure S3a. Emission fluorescence spectra for ThT assays (native pH 4). The inset shows the dependence of the position of the maxima, λ_0, on the concentration of peptide: (a) Smoc-Ala, (b) Smoc-Phe, (c) Smoc-Arg.

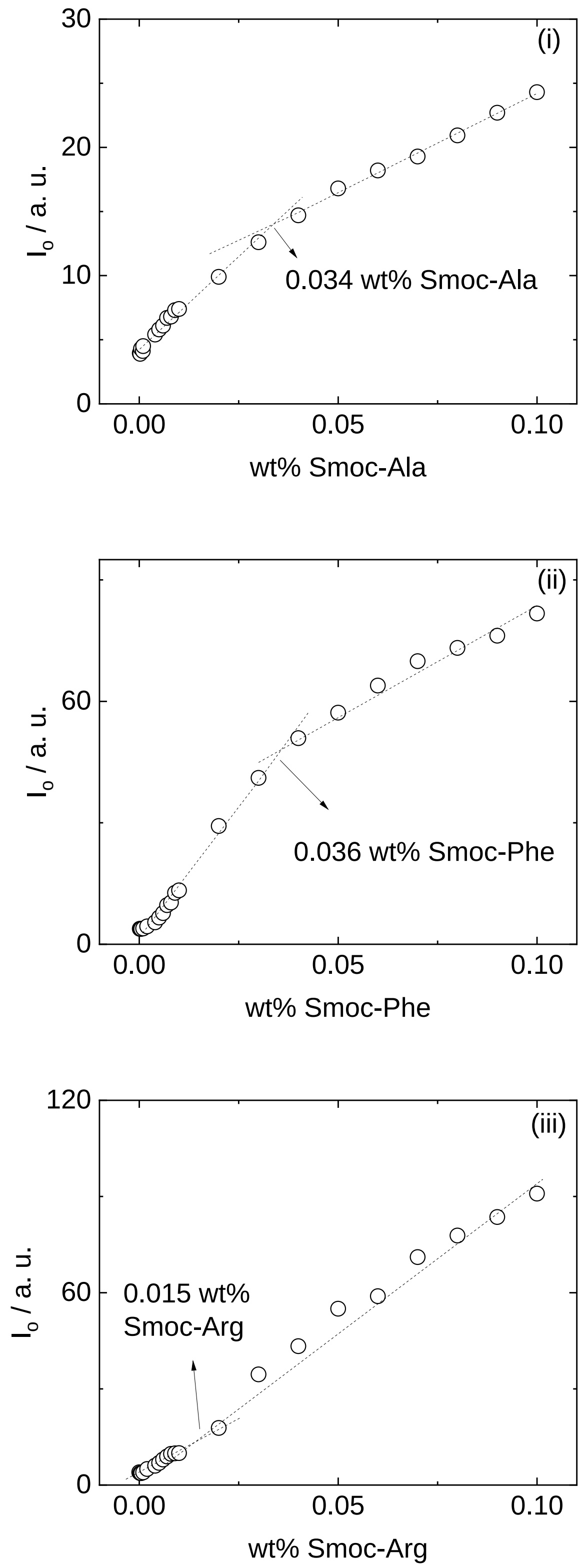

Figure S3b. Intensity of ThT fluorescence emission peak (I_0, SI Fig.S3a) vs peptide concentration at native pH for (a) Smoc-Ala, (b) Smoc-Phe, (c) Smoc-Arg.

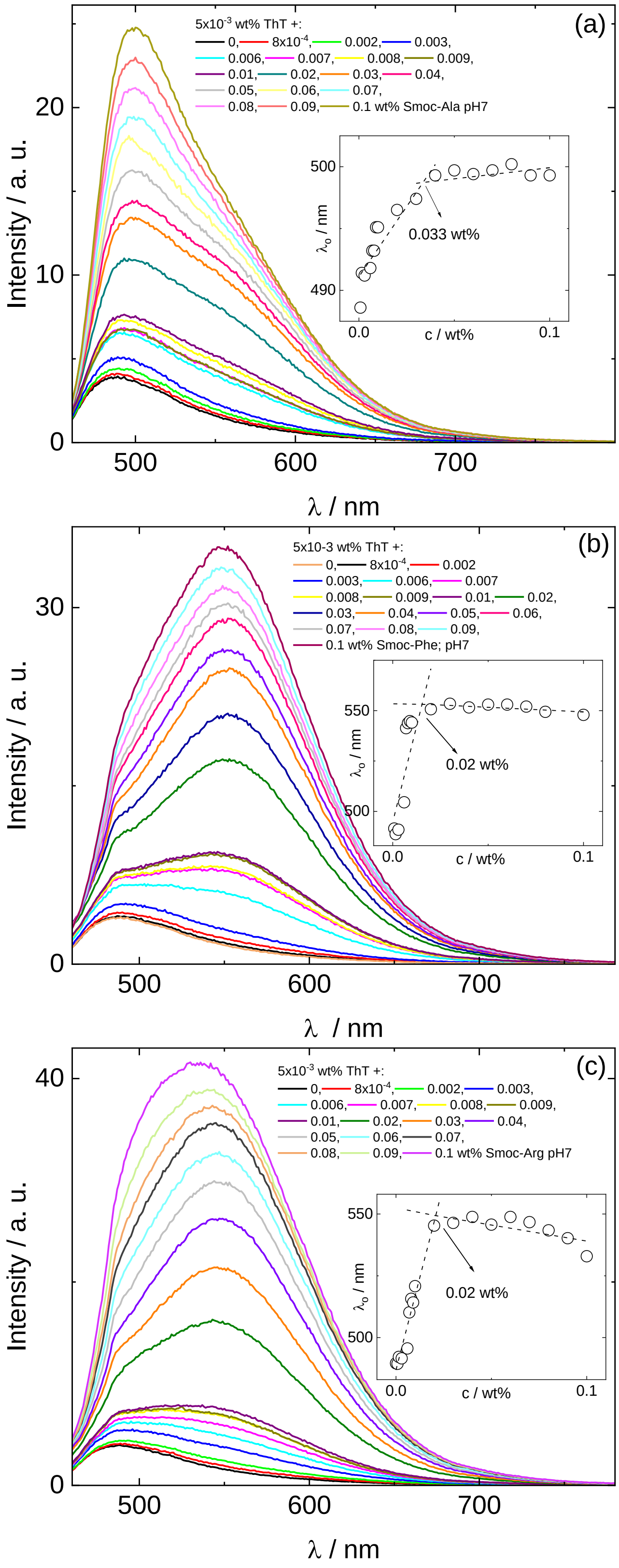

Figure S4. Emission fluorescence spectra for ThT assays at pH 7. The inset shows the dependence of the position of the maxima, λ_o, on the concentration of peptide: (a) Smoc-Ala, (b) Smoc-Phe, (c) Smoc-Arg.

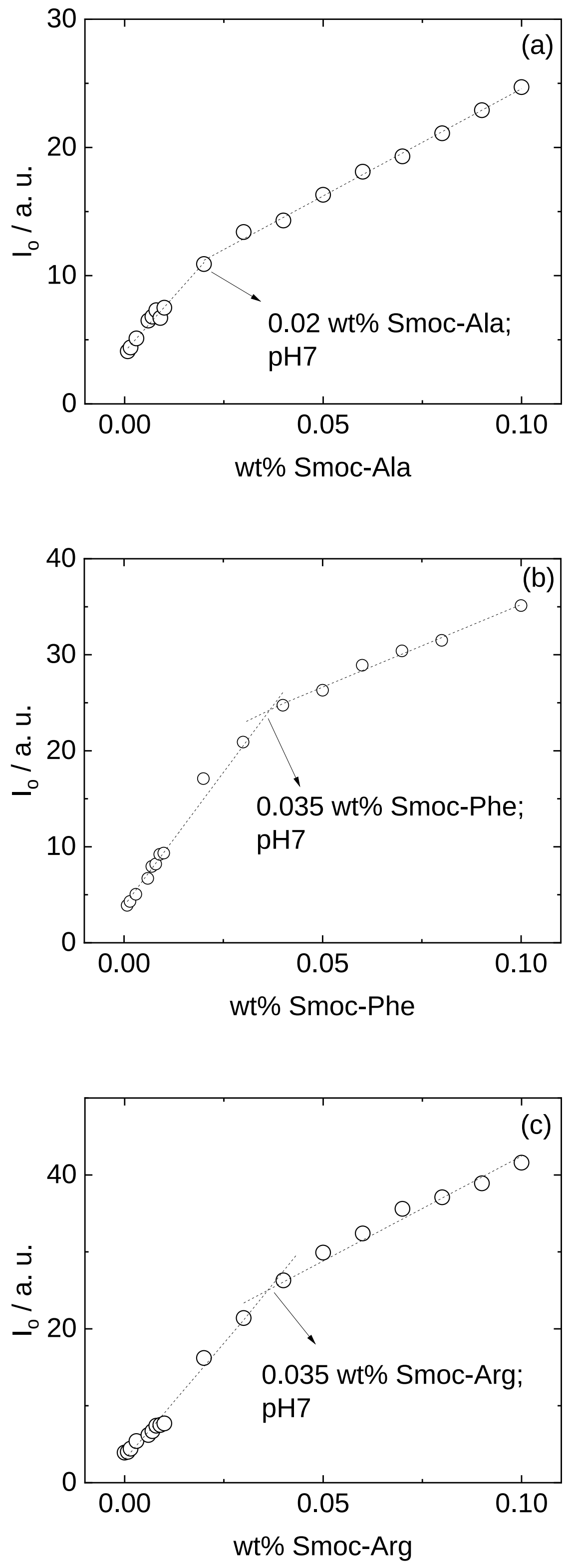

Figure S5. Intensity of ThT fluorescence emission peak (I_0, SI Fig.S4) vs peptide concentration at pH 7: (a) Smoc-Ala, (b) Smoc-Phe, (c) Smoc-Arg.

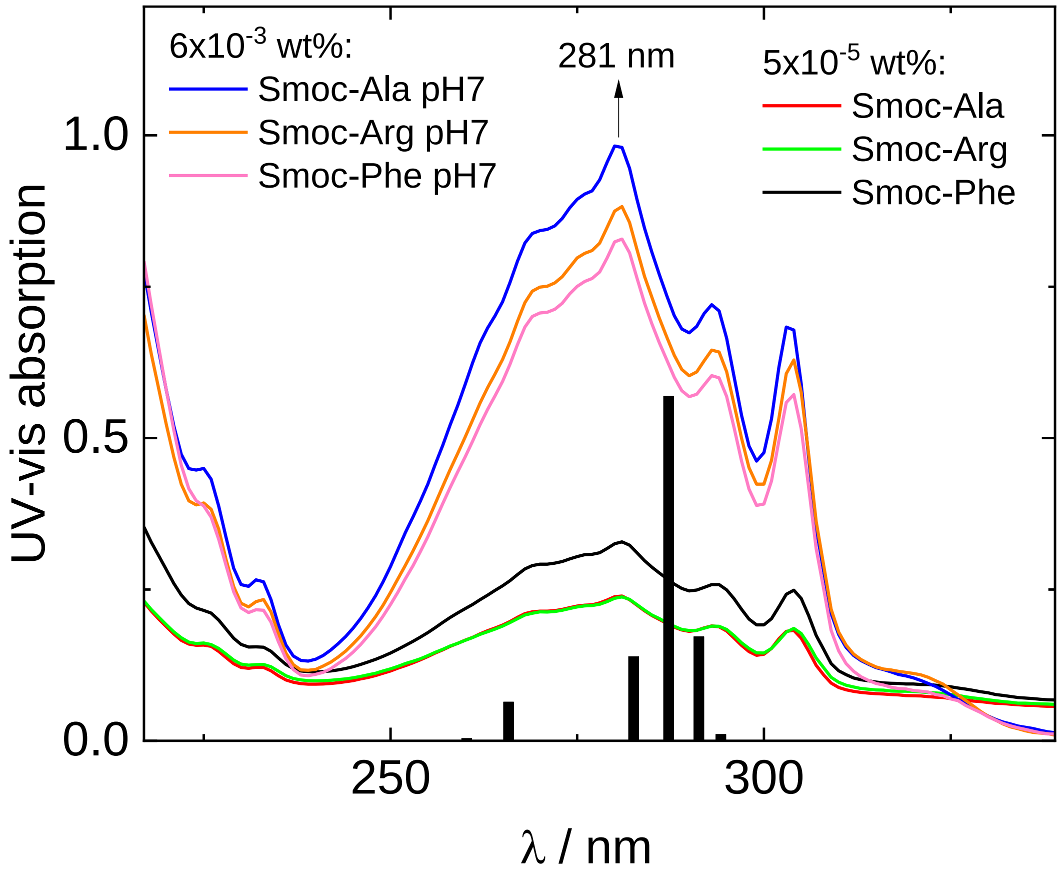

Figure S6. UV-vis absorption spectra of Smoc-amino acids: lines measured data, bars are DFT calculated band positions/strengths for Smoc-Ala. Data for sample dissolved in water or at pH7.

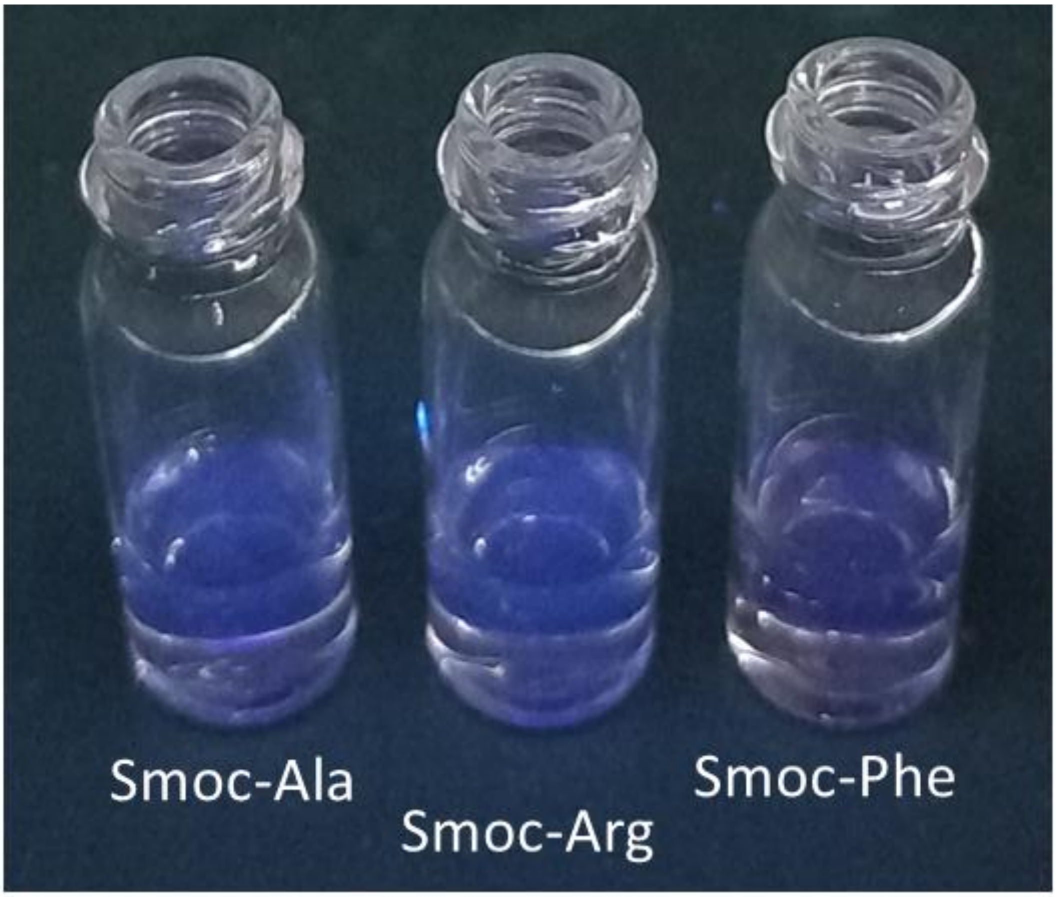

Figure S7. Fluorescence images obtained for samples at a concentration below the CAC (0.01 wt%), using a λ = 254 nm UV lamp.

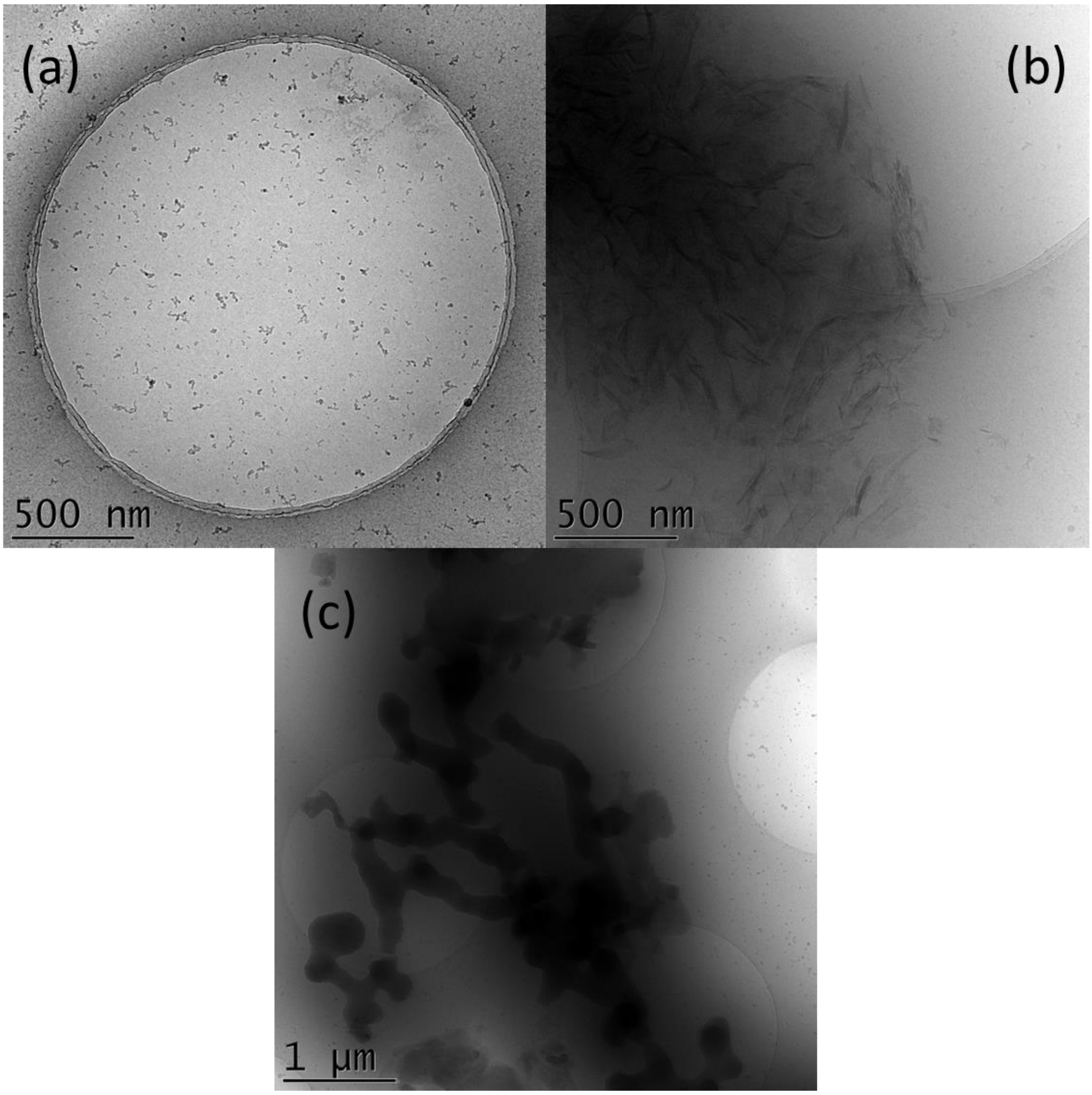

Figure S8. Additional Cryo-TEM images (native pH 4). (a) 1 wt% Smoc-Ala, (b) 1 wt% Smoc-Phe, (c) 0.5 wt% Smoc-Arg.

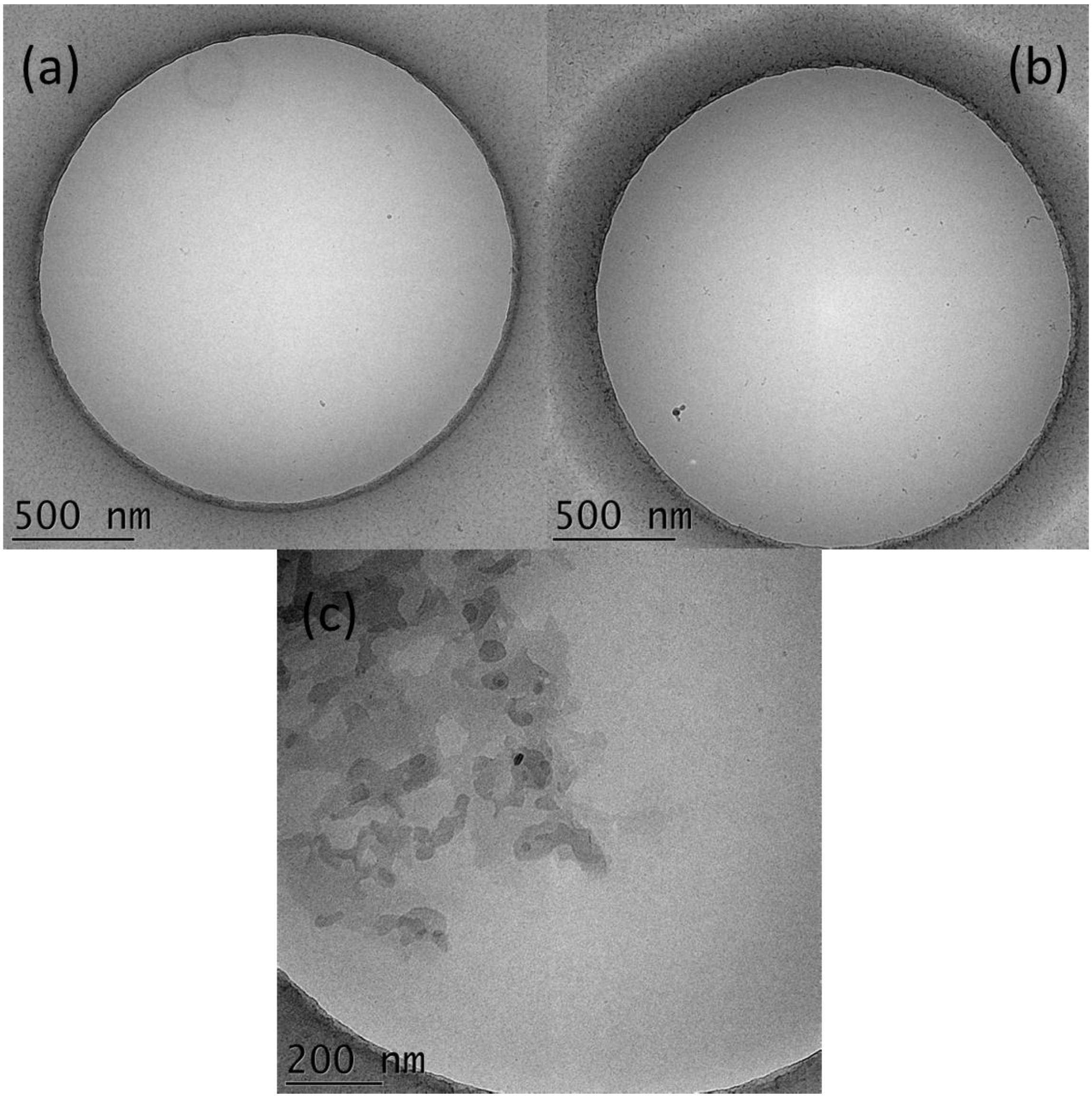

Figure S9. Representative cryo-TEM images (pH 7). (a) 1 wt% Smoc-Ala, (b) 1 wt% Smoc-Phe, (c) 0.5 wt% Smoc-Arg.

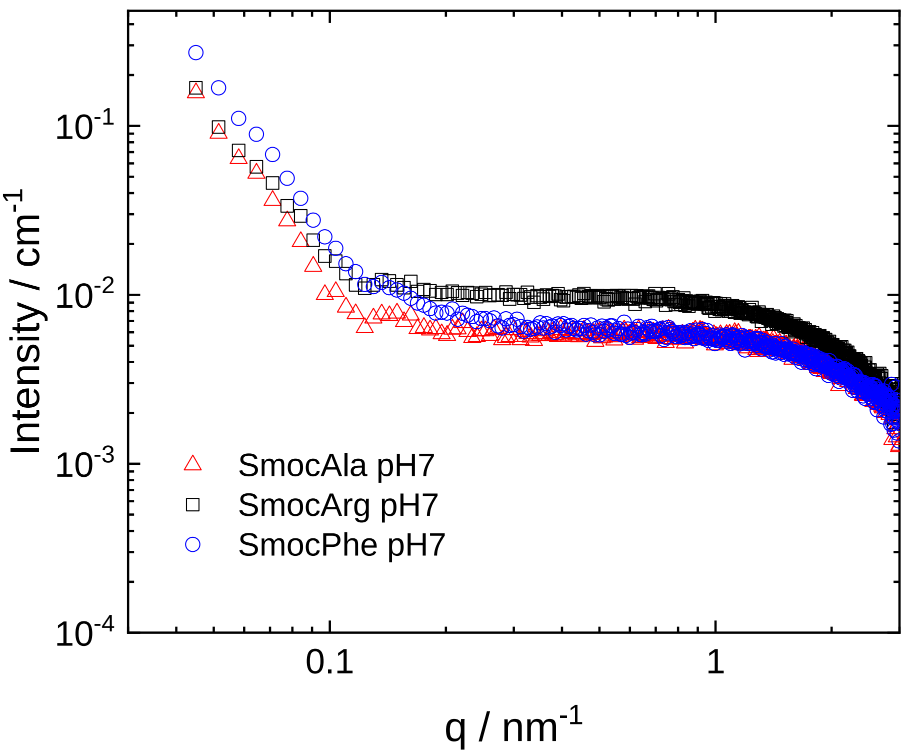

Figure S10. SAXS data for 1 wt% solutions at pH 7.

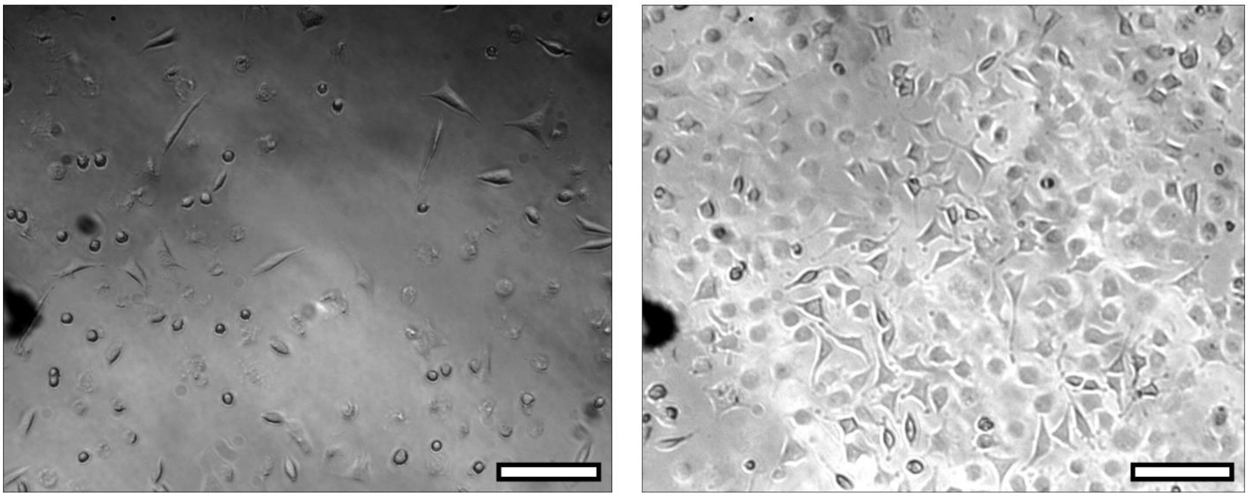

Figure S11. Optical microscopy images of cells after 72 h in the presence of Smoc-Ala at the concentrations indicated. Left: 0.1 wt%, right 0.0625 wt%. Scale bars: 100 µm.

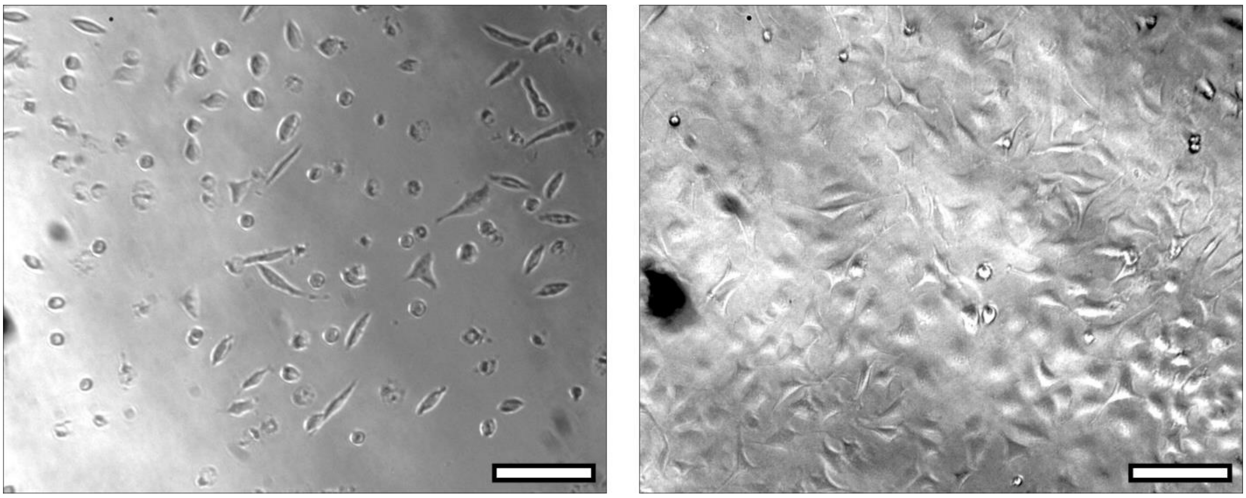

Figure S12. Optical microscopy images of cells after 72 h in the presence of Smoc-Phe at the concentrations indicated. Left: 0.1 wt%, right 0.0625 wt%. Scale bars: 100 µm.

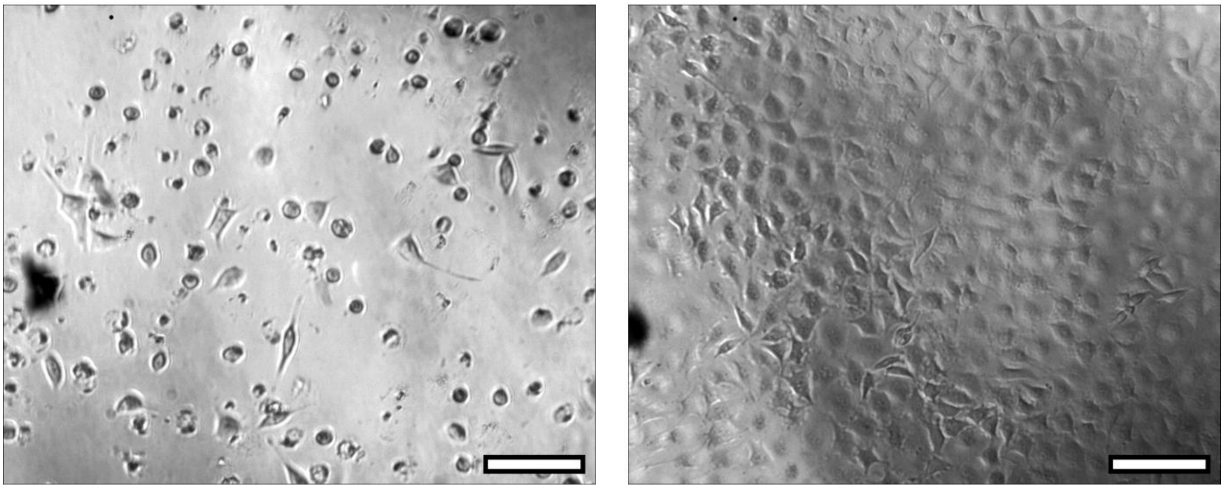

Figure S13. Optical microscopy images of cells after 72 h in the presence of Smoc-Arg at the concentrations indicated. Left: 0.1 wt%, right 0.0625 wt%. Scale bars: 100 µm.

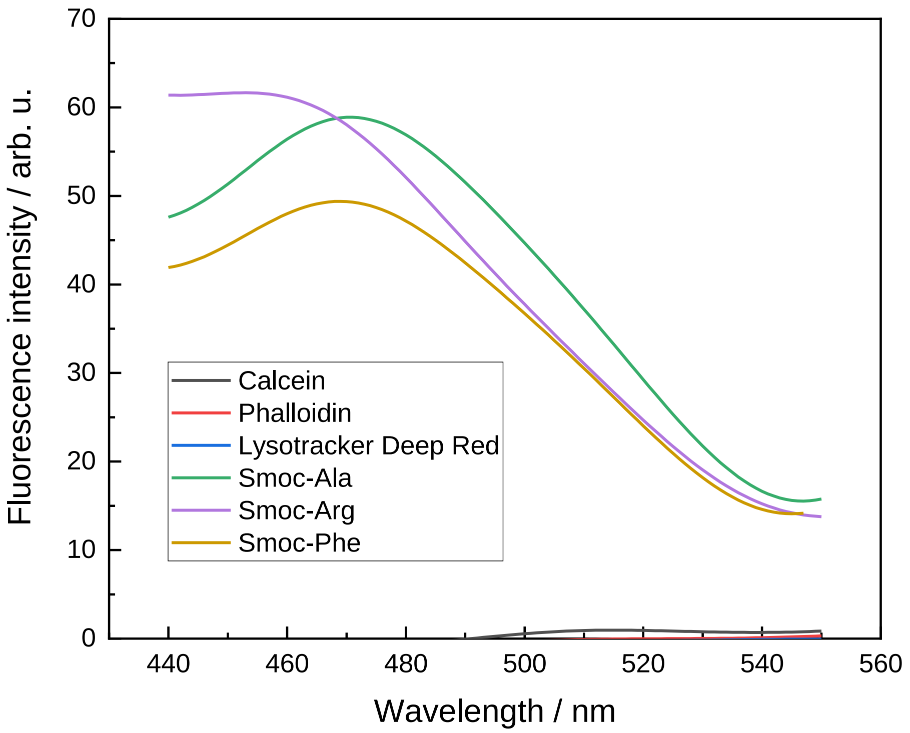

Figure S14. Fluorescence assay of the Smoc-amino acids (0.05 wt% concentration), phalloidin-Texas red, calcein and Lysotracker Deep Red under excitation at λ = 405 nm. The bandwidth of fluorescence set for the emission is the same as the confocal detector for this specific excitation.

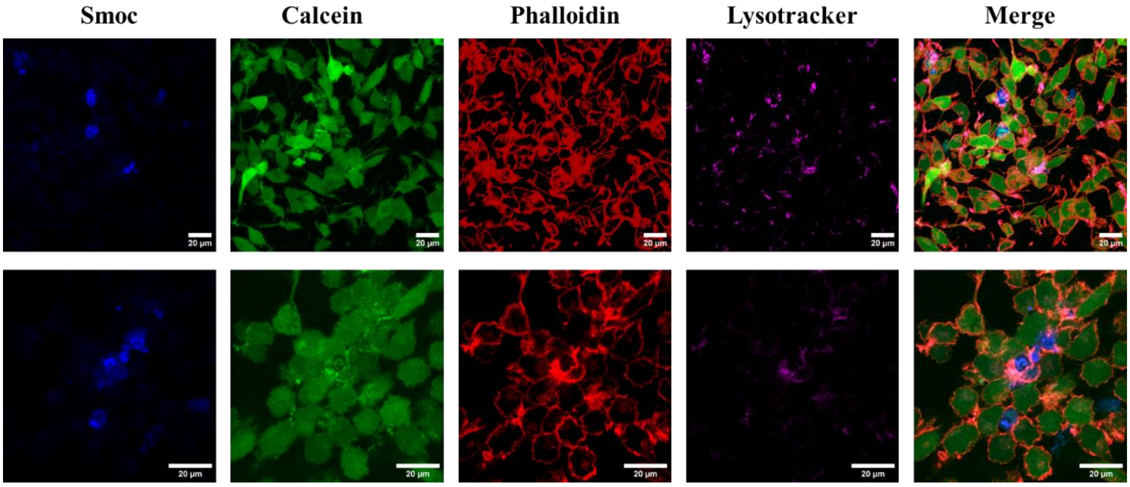

Figure S15. confocal microscopy of L929 cells incubated with 0.05 wt% Smoc-Ala in DMEM for 72 hours. The blue channel shows Smoc fluorescence, the green channel shows calcein fluorescence, the cytoskeleton stained with phalloidin Texas red is represented in red and the endosomal system is coloured in magenta by Lysotracker deep red. The last panel of each row is a merged image of all 4 channels.

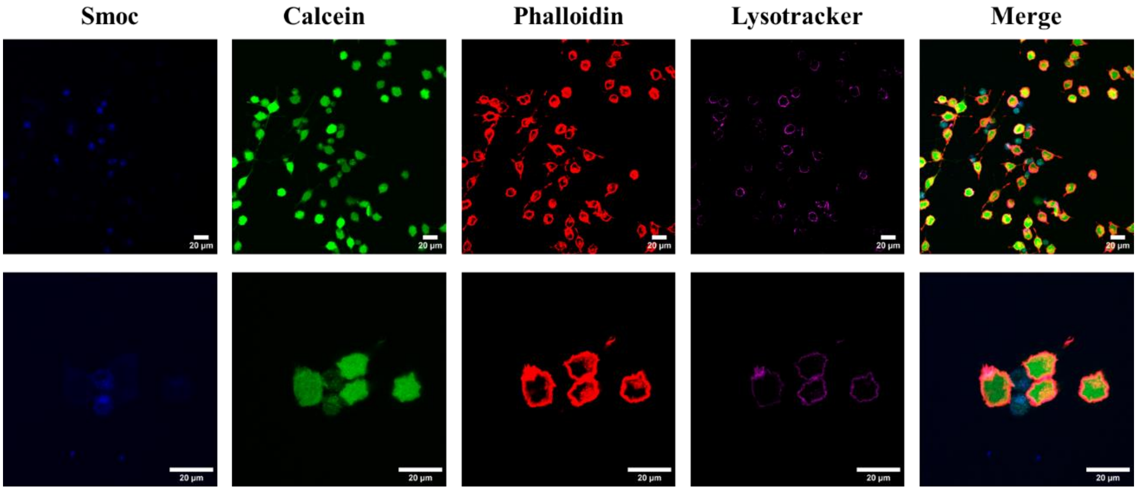

Figure S16. confocal microscopy of L929 cells incubated with 0.05 wt% Smoc-Phe in DMEM for 72 hours. The blue channel shows Smoc fluorescence, the green channel shows calcein fluorescence, the cytoskeleton stained with phalloidin Texas red is represented in red and the endosomal system is coloured in magenta by Lysotracker deep red. The last panel of each row is a merged image of all 4 channels.

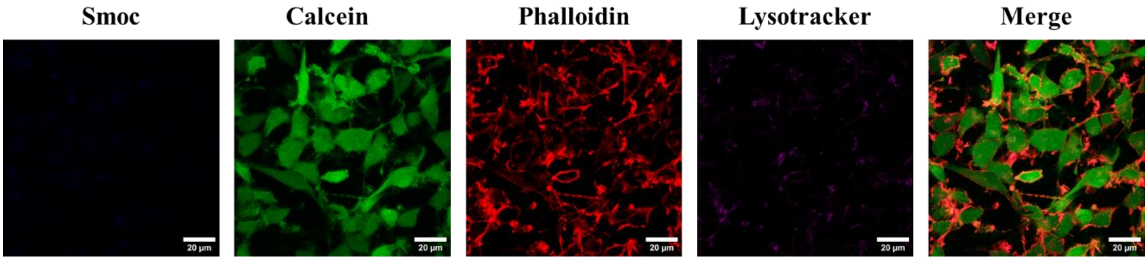

Figure S17. confocal microscopy of L929 cells incubated only in DMEM for 72 hours. The blue channel shows Smoc fluorescence, the green channel shows calcein fluorescence, the cytoskeleton stained with phalloidin Texas red is represented in red and the endosomal system is coloured in magenta by Lysotracker deep red. The last panel is a merged image of all 4 channels.

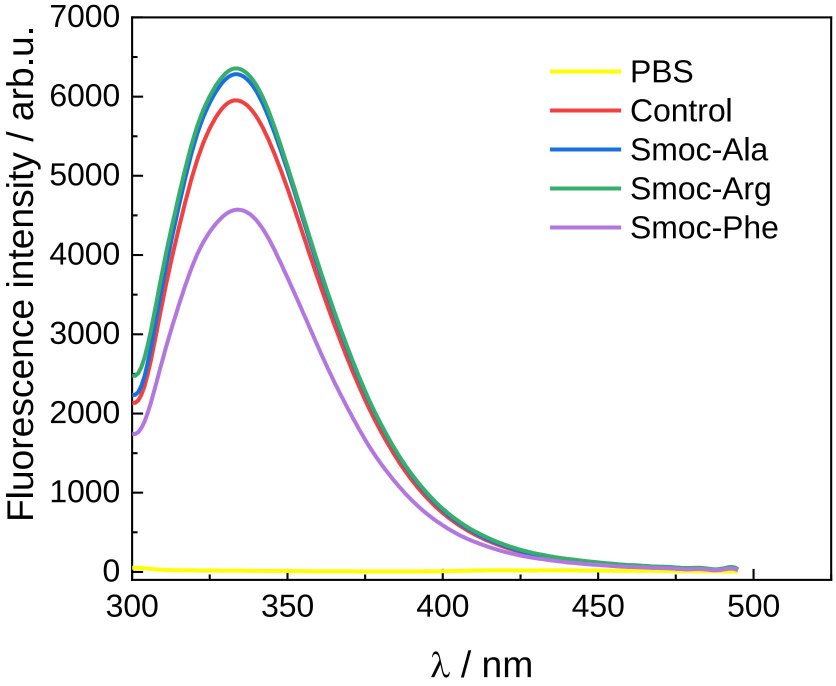

Figure S18. Fluorescence measurements for L929 fibroblast cells incubated with Smoc-amino acids (0.05 wt% solutions of Smoc samples).

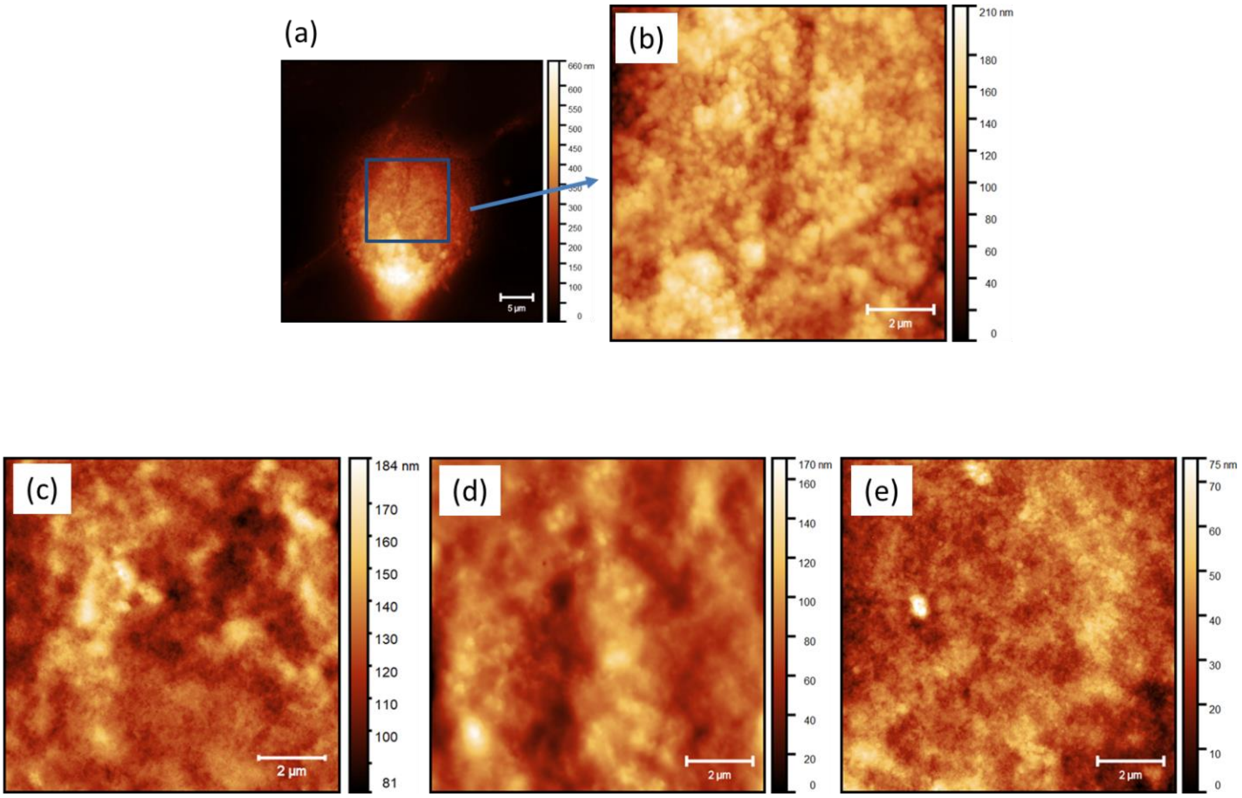

Figure S19. Images showing cell surfaces in the absence/presence of Smoc-amino acids. (a) L929 fibroblast control showing typical enlargement area, (b) Enlarged region of L929 fibroblast control, (c) L929 fibroblast exposed to Smoc-Ala, (d) L929 fibroblast exposed to Smoc-Phe, (e) L929 fibroblast exposed to Smoc-Arg.

Table S1. Parameters extracted from the fitting of the SAXS data in Figure 4, using generalized Gaussian coil along with power law intensity profile. Fitted using SASfit.[1-2]

	**1 wt% Smoc-Ala[a]**	**1 wt% Smoc-Phe[b]**	**1% Smoc-Arg[c]**
R_g/ nm	0.62	0.64	0.62
ν	0.17	0.15	0.17
I_0/ cm^{-1}	3.7×10^{-3}	3.9×10^{-3}	8.4×10^{-3}
I_1/ cm^{-1}		2.62×10^{-4}	1.87×10^{-6}
x		3.74	3.99
BG	1.5×10^{-3}	1.1×10^{-3}	9.6×10^{-4}

Key:
[a] **Generalized Gaussian Coil:** R_g, radius of gyration, ν Flory exponent, I_0 forward intensity.
[b,c] **Generalized Gaussian Coil + Low q sloping intensity:** R_g, radius of gyration, ν Flory exponent, I_0 forward intensity, Low q sloping intensity ($I_1 q^{-x}$), I_1 intensity , x exponent.
[c] The parameters for the generalized Gaussian coil were set to be the same as those for Smoc-Ala.
All fits: **Constant Background** BG.

References

(1) Bressler, I.; Kohlbrecher, J.; Thünemann, A. F., SASfit: a tool for small-angle scattering data analysis using a library of analytical expressions. *J. Appl. Cryst.* **2015,** *48*, 1587-1598.
(2) Kohlbrecher, J.; Bressler, I., Updates in SASfit for fitting analytical expressions and numerical models to small-angle scattering patterns. *J. Appl. Cryst.* **2022,** *55*, 1677-1688.
